# Enhanced Redox Electrocatalysis in High-Entropy Perovskite Fluorides by Tailoring *d*–*p* Hybridization

**DOI:** 10.1007/s40820-023-01275-3

**Published:** 2023-12-18

**Authors:** Xudong Li, Zhuomin Qiang, Guokang Han, Shuyun Guan, Yang Zhao, Shuaifeng Lou, Yongming Zhu

**Affiliations:** 1 Department of Applied Chemistry, Harbin Institute of Technology at Weihai, Weihai, 264209 People’s Republic of China; 2 MIIT Key Laboratory of Critical Materials Technology for New Energy Conversion and Storage, School of Chemistry and Chemical Engineering, Harbin Institute of Technology, Harbin, 150001 People’s Republic of China

**Keywords:** Lithium–oxygen batteries, KCoMnNiMgZnF_3_-HEC perovskite fluoride, Entropy effect, Catalytic kinetics, *d–p* orbital hybridization

## Abstract

High-entropy catalysts featuring exceptional properties are, in no doubt, playing an increasingly significant role in aprotic lithium-oxygen batteries. Despite extensive effort devoted to tracing the origin of their unparalleled performance, the relationships between multiple active sites and reaction intermediates are still obscure. Here, enlightened by theoretical screening, we tailor a high-entropy perovskite fluoride (KCoMnNiMgZnF_3_-HEC) with various active sites to overcome the limitations of conventional catalysts in redox process. The entropy effect modulates the *d*-band center and d orbital occupancy of active centers, which optimizes the *d*–*p* hybridization between catalytic sites and key intermediates, enabling a moderate adsorption of LiO_2_ and thus reinforcing the reaction kinetics. As a result, the Li–O_2_ battery with KCoMnNiMgZnF_3_-HEC catalyst delivers a minimal discharge/charge polarization and long-term cycle stability, preceding majority of traditional catalysts reported. These encouraging results provide inspiring insights into the electron manipulation and *d* orbital structure optimization for advanced electrocatalyst.

**Supplementary Information:**

The online version contains supplementary material available at 10.1007/s40820-023-01275-3.

## Highlights

- The tailored KCoMnNiMgZnF_3_-HEC cathode delivers extremely high discharge capacity (22,104 mAh g^−1^), outstanding long-term cyclability (over 500 h), preceding majority of traditional catalysts reported.
- Entropy effect of multiple sites in KCoMnNiMgZnF_3_-HEC engenders appropriate regulation of 3*d* orbital structure, leading to a moderate hybridization with the *p* orbital of key intermediate.
- The homogeneous nucleation of Li_2_O_2_ is achieved on multiple cation site, contributing to effective mass transfer at the three-phase interface, and thus, the reversibility of O_2_/Li_2_O_2_ conversion.

**Supplementary Information:**

The online version contains supplementary material available at 10.1007/s40820-023-01275-3.

## Graphical abstract

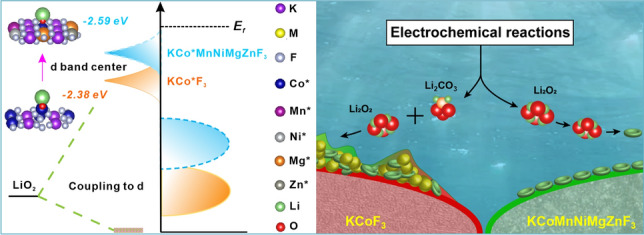

## Introduction

Rechargeable lithium–oxygen battery (LOB) has drawn intriguing attraction owing to its ultrahigh theoretical energy density (3500 Wh kg^−1^), which can be expected to become ultimate candidate to replace current state-of-the-art lithium-ion battery [1]. Nevertheless, LOB suffers from low round-trip efficiency and poor cyclability, accompanied with large polarization. Those intractable issues mainly lie in the sluggish oxygen reduction reaction (ORR) and oxygen evolution reaction (OER), closely related to the complex O_2_/Li_2_O_2_ redox chemistry in the air cathodes [2]. The reversibility of Li_2_O_2_ deposition/decomposition directly determines the performance of LOB, highlighting the necessity of ameliorating reaction kinetics through constructing well-tailored catalysts. It is widely accepted that catalyst activity depends substantially on the adsorption/desorption of intermediates on surface-active sites, which can be modulated via tuning orbital occupancy of active centers [3–5]. Various elaborate catalysts with *d*-block transition metals, as LiO_2_-affinity regulators, have been rapidly explored in recent years to boosting catalytic conversion of O_2_/Li_2_O_2_ in LOBs [6–10]. Despite the considerable optimization in catalytic kinetics, the functionality and availability of such catalysts still fall short of expectations, mainly due to the limited number of accessible active sites.

High entropy compounds (HECs), a new frontier in catalysis with broad chemical space tunability, have shown particularly high catalytic activities compared to conventional mono- and bimetallic nanocrystals [11, 12]. Infinite elemental combinations enrich the diversities of active sites and local electronic structures, which provides more possibilities for tailoring of functional properties, and thus expands a wide design platform for catalysts with desirable activity, durability, and effectivity. Theoretically, synergistic effect and interaction of dissimilar species can enhance site-to-site electron transfer, allowing simultaneous stabilization of reaction intermediates with moderate binding energies in multi-steps/electron/phase redox conversions of LOB electrochemistry. A majority of works have been dedicated to the exploration of catalytic performance for HECs, and however, the understanding of activity origins and correlations between intrinsic electronic structure and reaction intermediates in HECs is greatly neglected [13–15]. Furthermore, the influence of multiple active sites on the nucleation/growth kinetics of Li_2_O_2_ in redox processes remains ambiguous, hindering the selection and rational design of HECs catalysts for LOBs.

Herein, a sophisticated KCoMnNiMgZnF_3_, and HEC-perovskite fluoride, was first introduced into LOBs to overcome the limitations of conventional catalysts in redox process. We systematically investigated the local electronic environments of cation sites and identified *d*-band-dependent preferences for the adsorption of reaction intermediates during catalysis. The density function theory (DFT) calculations revealed that the *d*-band center and *d* orbital occupancy of active centers can be regulated by entropy effect, which engenders a favorable *d*-*p* orbital hybridization between multiple active cations in HEC and LiO_2_. This moderate interaction grants KCoMnNiMgZnF_3_-HEC the enhanced catalytic kinetics, effectively lowering the redox energy barrier. Experimental analysis combined with COMSOL multiphysics demonstrated that the multiple active sites in KCoMnNiMgZnF_3_-HEC moiety served as nucleation seeds synergistically facilitate the homogeneous formation of Li_2_O_2_, which in favor of the mass transfer, leading to an impressive electrochemical performance of LOB. The LOB with KCoMnNiMgZnF_3_-HEC catalyst achieves a very low overall discharge/charge overpotential (0.7 V), extremely high discharge capacity (22,104 mAh g^−1^ at a current density of 200 mA g^−1^) and outstanding long-term cyclability (over 500 cycles at a current density of 1000 mA g^−1^ with a capacity limitation of 1000 mAh g^−1^), which is superior to that of the current traditional catalysts. Our work suggests the possibility of manipulating the reaction kinetics and nucleation/growth mechanisms of Li_2_O_2_ by the unique entropy effect of multiple sites in HEC, providing guidance to the rational design of efficient active sites for LOBs.

## Experimental Section

### Materials

Cobalt chloride hexahydrate (CoCl_2_·6H_2_O, AR), nickel(II) chloride hexahydrate (NiCl_2_·6H_2_O, AR), manganese chloride tetrahydrate (MnCl_2_·4H_2_O, AR), zinc chloride (ZnCl_2_, AR), Potassium fluoride dihydrate (KF·2H_2_O, AR), polyvinyl pyrrolidone (PVP-K29-32, AR) and ethylene glycol (C_2_H_6_O_2_, AR) were supplied from Shanghai Aladdin Biochemical Technology Co., Ltd. (China). The CR2032 cell, glass fiber separator (GF/B), lithium bis(trifluoromethanesulphonyl)imide (LiN(CF_3_SO_2_)_2_), ≥ 99.9 wt%), tetraethylene glycol dimethyl ether (C_10_H_22_O_5_, ≥ 99.5%(GC)), Ketjen Black (KB EC-600JD), lithium foil and carbon paper (H060) were purchased by Aisim (Shenzhen) Technology Co., Ltd. (China).

### Preparation of GO, HCPA and GO/HCPA Nanocomposite Papers

#### Preparations of the KCoF_3_, KCoMnNiF_3_, KCoMnNiMgF_3_ and KCoMnNiZnF_3_ Samples

Perovskite fluorides were synthesized accordant with reference as reported, but with some modifications here. Taking the KCoMnNiZnF_3_-HEC as a representative, 0.8 mmol CoCl_2_·6H_2_O, 0.8 mmol NiCl_2_·6H_2_O, 0.8 mmol MnCl_2_·4H_2_O, 0.8 mmol MgCl_2_, 0.8 mmol ZnCl_2_, 10 mmol KF·2H_2_O and 0.4 g PVP-K29-32 were added into in 72 mL ethylene glycol solvent. After dissolving completely, the mixture was treated solvent thermally in a teflon-lined stainless-steel autoclave at 180 °C for 12 h in a homogeneous reactor, and allowed to cool-down naturally. Subsequently, the sample was washed with absolute alcohol, repeated several times until the solution clean up. Finally, the compound was dried in vacuum at 80 °C, obtaining the KCoMnNiZnF_3_-HEC material. As for other samples, the synthetic process complied with the above procedure, according to their metallic element ratios with the total molar ratio of 4 mmol.

#### Materials Characterizations

The X-ray diffraction (XRD) was performed on a Rigaku D/max-rb diffractometer equipped with Cu Kα radiation (*λ* = 1.5406 Å, 40 kV, 20 mA). Transmission electron microscopy (TEM) and high-angle annular dark-field scanning transmission electron microscopy (HAADF-STEM) images were recorded on JEOL JEM2100 microscope operated at 200 kV. Energy dispersive spectroscopy (EDS) was conducted on NORAN system 7 equipped with TEM. PerkinElmer Optima 5300DV ICP-OES system was used to determine the mass fraction of elements. The X-ray photoelectron spectroscopy (XPS) was carried out on Physical Electronics PHI model 5700 instrument with Al Kα radiation. The XAFS spectra were measured on Super Photon ring-8 of the Japan Synchrotron Radiation Research Institute. The electron paramagnetic resonance (EPR) spectroscopy was determined with JEOL JES-X-320 operating at X-band frequency of 9.79 GHz. The Co K-edge XANES data were accounted in transmission mode with Co foil, CoO and Co_3_O_4_ as references. The obtained EXAFS data was processed based on the standard routine utilizing the Athena software packages. The EXAFS spectra were obtained by deducing the post-edge background from the overall absorption and then normalizing in respect of the edge-jump step. Raman spectroscopy (Raman) was were collected on Invia-Reflex of Renishaw with wavelength of 532 nm. The scanning electron microscopy (SEM) was conducted on Helios Nanolab 600i at 20 kV. The in-situ differential electrochemical mass spectrometry was tested at HPR20-EGA.

#### Electrochemical Measurements

*Li–O*_*2*_* cell assembly and testing* the perovskite fluorides catalysts (30%) and Ketjen Black EC-600JD (KB, 50%) were uniform mixed with polyvinyl pyrrolidone (PVP, 20%) binder in mixture (isopropanol/water = 1:3) to form a homogeneous ink, which was sprayed on a piece of carbon paper (*D* = 14 mm). After vacuum drying at 80 °C for 12 h, the O_2_-electrodes were obtained. The electrolyte adopted 1 M lithium bis(trifluoromethane)sulfonimide (LiTFSI) in triethylene glycol dimethyl ether (TEGDME). The anode and separator used lithium foils and Whatman GF/D glass fibers, respectively. The 2032-tyoe coin cells were assembled in an argon-filled glovebox with oxygen and moisture contents below 0.1 ppm. The galvanostatic charge–discharge performances were conducted on a battery test system (LAND, China) at different current densities in the voltage scope of 2.0–4.5 V versus Li/Li^+^, or in a capacity limitation. The cyclic stability measures were conducted under a limited capacity. All the evaluation tests mentioned above were carried out at room temperature.

#### Computational Calculations and Simulations

*The density functional theory (DFT) calculations* the DFT were conducted on the Vienna ab initio simulation package (VASP). The models were constructed based on KCoF_3_ (110) (17.7 Å × 8.35 Å × 11.25 Å), and the vacuum depth was set to 20 Å. The 3*d*/4*s* electrons of Co, Ni, Mn and Zn atoms, 2*s*/2*p* electrons for F, 2*s*/2*p*/3*s* electrons for Mg, and 4*s*/3*p*/4*s* electrons for K were treated as valence electrons. The generalized gradient approximation (GGA) were used as the gradient correction function (Perdew–Burke–Ernzerhof (PBE). The interaction between valence electrons and core electrons was described by the plane-mode conserved pseudopotential (PAW). The dispersion interactions were described by Grimme’s DFT-D3 methodology. The 2 × 3 × 1 grid points were performed using the Monkhorst–Pack method in the Brillouin region with plane wave truncation energy of 400 eV. In the optimization process, the energy convergence accuracy was set to 1.0 × 10^–5^ eV atom^−1^, and the structure relaxation was conducted until the maximum force on each atom below 0.02 eV Å^−1^.

The energy of adsorption for reaction intermediates was calculated as follows:

1 $$E_{{{\text{adsorption}}}} = E_{{\text{t}}} - E_{a} - E_{b}$$

where *E*_t_ is the total energy of the adsorbed system, $E_{a}$ and $E_{b}$ represent the total energy of free species and bare surface, respectively.

The phase diagram of the discharge products is built based on the adsorption energy calculation. The SSLOB ORR/OER reaction free energy were determined by Norskov’s calculation method.

*Computational simulations* the simulations were conducted on the COMSOL Multiphysics 6.0 software, and the operational details were shown in support information.

## Results and Discussion

### Activity Origins of HECs

**Fig. 1:**
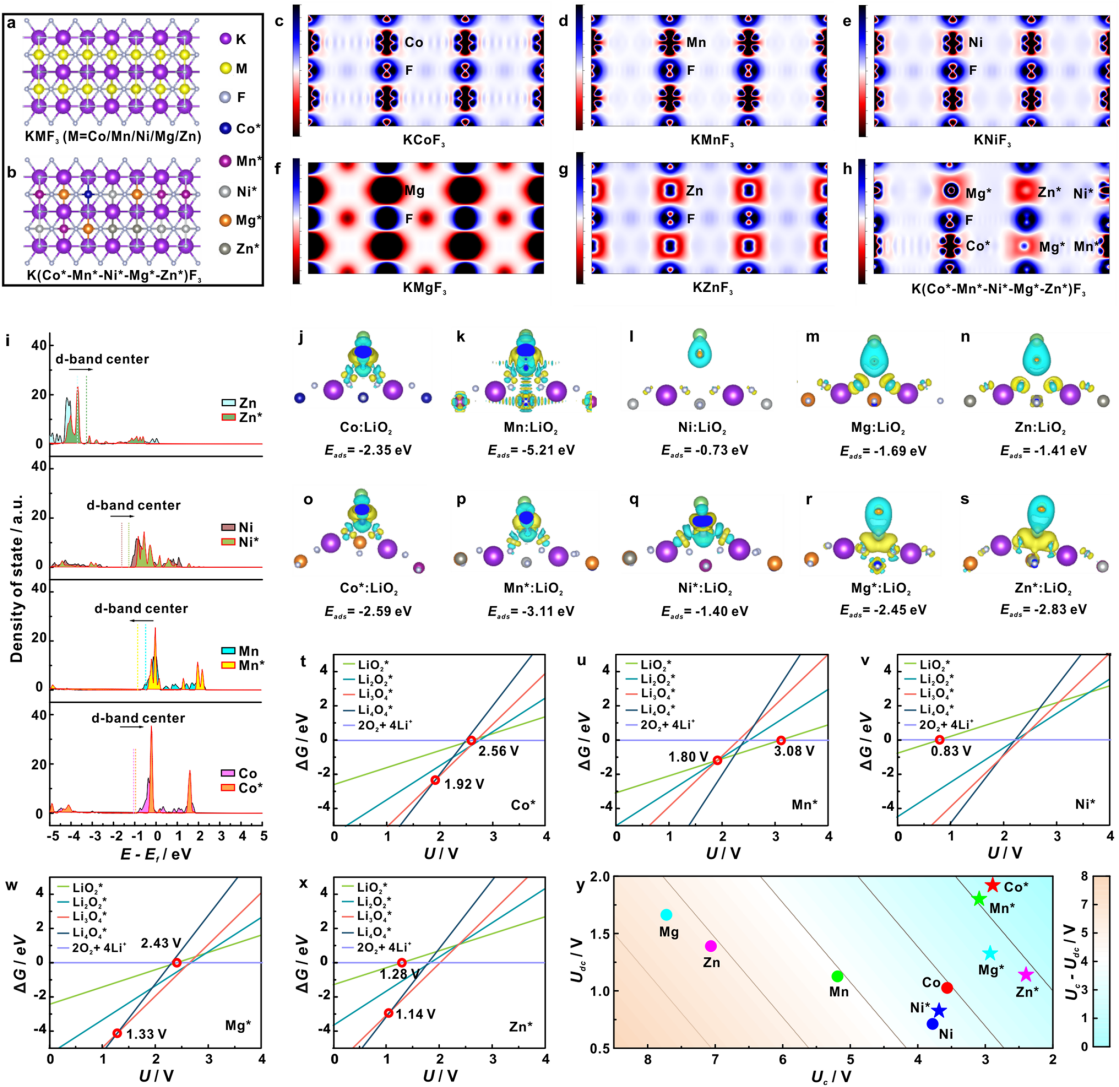
The optimized structural models of **a** KMF_3_ and **b** KCoMnNiMgZnF_3_-HEC. Cross-section of the charge density difference diagrams of **c** KCoF_3_, **d** KMnF_3_, **e** KNiF_3_, **f** KMgF_3_, **g** KZnF_3_, **h** KCoMnNiMgZnF_3_-HEC. **i** PDOS and corresponding calculated d-band centers of Co 3*d* in different models. Optimized structures and the corresponding binding energy of intermediate LiO_2_ on the sites of **j** Co in KCoF_3_, **k** Ni in KNiF_3_, **l** Ni in KNiF_3_, **m** Mg in KMgF_3_, **n** Zn in KZnF_3_, and **o** Co*, **p** Mn*, **q** Ni*, **r** Mg* and **s** Zn* in KCoMnNiMgZnF_3_-HEC. The phase diagram of possible intermediates and discharge products on the sites of **t** Co*, **u** Mn*, **v** Ni*, **w** Mg* and **x** Zn* in KCoMnNiMgZnF_3_-HEC. **y** The contours of predicted catalytic activity for different sites in various models

To reveal the influence of local electronic structure in HECs on the catalytic conversion of O_2_/Li_2_O_2_, a series of unitary KMF_3_ (M = Co, Mn, Ni, Mg, Zn) and quinary KCoMnNiMgZnF_3_-HEC models were established for DFT calculations (Fig. 1a, b). Electron localization functions (Fig. 1c-h,) and Bader charge (Table S1) elucidate the electron density redistribution of interfacial metal atoms in HEC, which modulates the *d*-band center, as evidenced by the projected density of states (PDOS, Fig. 1i). Figures 1j–s and S1 illustrate the diagrams of charge density difference for the LiO_2_ adsorbed on different sites and the corresponding adsorption energy (Δ*E*_*a*_), in that the blue area and yellow area presents the lost electron and the gained electron, respectively. Clearly, abundant electron transfers occur on metal sites, manifesting the strong electronic interaction between reaction sites and LiO_2_. It’s worth noting that the Co*, Mn*, Ni*, Mg*, Zn* site in KCoMnNiMgZnF_3_-HEC achieves more appealing Δ*E*_*b*_ of − 2.59, − 3.11, − 1.40, − 2.45, − 2.83 eV, respectively, compared to those in KMF_3_ candidates, enabling a moderate binding strength of LiO_2_. In fact, the O_2_/Li_2_O_2_ conversion in LOB system involves the *d*–*p* orbital hybridization between active site and key intermediate LiO_2_. This interaction is related to the occupancy state of antibonding orbitals, which depends on the energy level of *d*-band, according to the *d*-band theory [8, 16, 17]. Since the antibonding states are always above the *d* states in terms of energy, the *d*-band center model would be a significant descriptor of the interaction between the catalyst and intermediate. Taking Co* adsorbed configuration as a representative, *d*-band center in HEC shows a slight upshift trend (Fig. 1i), implying the less filling of the antibonding states, leading to an elevated coupling of LiO_2_ (Fig. 1j, o). Therefore, it is believed that the entropy effect manipulates the local electron configuration and the *d* orbital electron filling, which endows multiple metal active sites in KCoMnNiMgZnF_3_-HEC with a favorable adsorbed energy of LiO_2_.

To profoundly understand the ORR/OER thermodynamics on multiple sites in HEC, we established the phase diagram of possible intermediates (Li_*x*_O_*y*_, *x* = 1, *y* = 2; *x* = 3, *y* = 4) and discharge products (Fig. 1t–x). The adsorption energy calculation is defined as Δ*E* = Δ*E*_0_ + *neU*, where *n*, *e*, and *U* represents the charge transfer number, electron charge, and electrode potential, respectively [18]. The formation of LiO_2_ is thermodynamically advantageous on Co*, Mn*, Ni*, Mg*, Zn* site when the discharge voltage is below 2.56, 3.08, 0.83, 2.43, 1.28 V, respectively. Notably, the LiO_2_ can maintain stability on Mn* site at potentials greater than 1.80 V, while Li_3_O_4_ dominates at potentials greater than 1.92, 1.33, 1.14 V on Co*, Mg*, Zn* site, respectively. These results substantiate the ability of multiple sites in HEC for stabilizing Li_2−*x*_O_2_ intermediates. The difference (Δ*U*) between minimum OER potential and maximum ORR potential (Fig. 1y), as a theoretical descriptor of catalytic activity, is predicted on the proposed models based on the Gibbs free energy of each endothermic step (Tables S2, S3). Apparently, Co*, Mn*, Ni*, Mg*, Zn* site in KCoMnNiMgZnF_3_-HEC shows a lower Δ*U* compared with that in KMF_3_, suggesting the enhanced reaction kinetics. Therefore, the appropriate electronic environment of the KCoMnNiMgZnF_3_-HEC triggered by entropy effect can realize the multi-active sites for moderate adsorption of key intermediates during the electrocatalysis, which maximizes the utilization of surface electroactivity, dramatically optimizing the reaction pathway.

### Synthesis and Structural Characterization

**Fig. 2:**
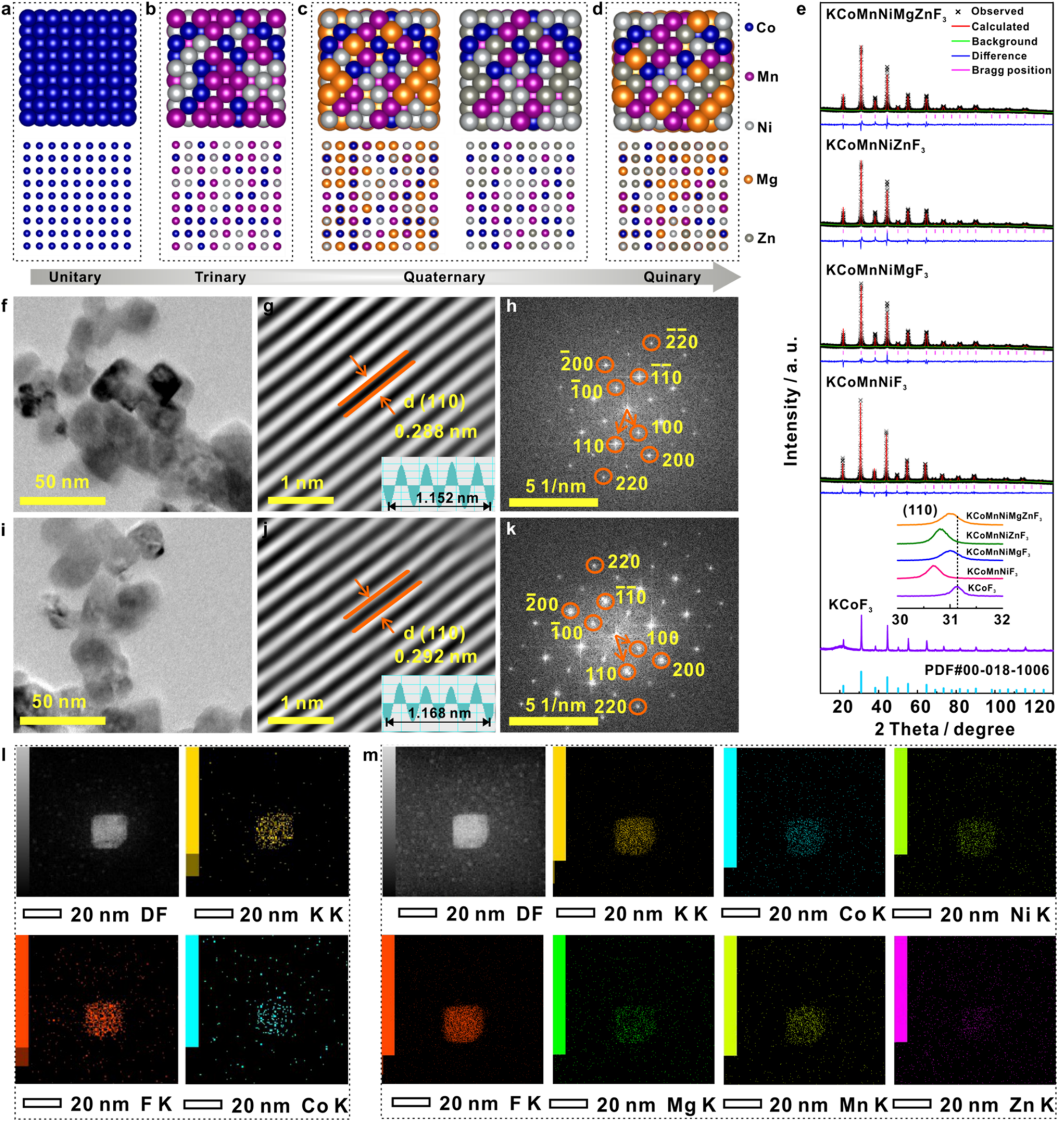
The structural schematic diagram perovskite fluorides for **a** unitary, **b** trinary, **c** quaternary and **d** quinary. **e** XRD patterns of KCoF_3_, KCoMnNiF_3_, KCoMnNiMgF_3_, KCoMnNiZnF_3_ and KCoMnNiMgZnF_3_-HEC with detailed rietveld refinements. TEM (**f**), the inversed fast Fourier transform (IFFT) image (**g**), and FFT pattern (**h**) of KCoF_3_. TEM (**i**), the IFFT image (**j**) and FFT pattern (**k**) of KCoMnNiMgZnF_3_-HEC. EDS mapping of **l** KCoF_3_ and **m** KCoMnNiMgZnF_3_-HEC

Inspired by the highlighted catalytic activity of KCoMnNiMgZnF_3_-HEC, we would like to shed light on the essentials of synergistic regulation mechanisms in HEC. Based on the DFT prediction, the model electrocatalysts, KCoF_3_, KCoMnNiF_3_, KCoMnNiMgF_3_, KCoMnNiZnF_3_, KCoMnNiMgZnF_3_-HEC were experimentally prepared through a one-pot solvothermal route, as illustrated in Fig. 2a–d. In XRD patterns with detailed Rietveld refinements (Fig. 2e), the KCoMnNiMgZnF_3_-HEC shares the same characteristics to that of KCoF_3_, suggesting the cubic perovskite structures. Obviously, the diffraction peak originating from the (110) plane slightly shift to lower angles after changing in the numbers of elements, demonstrating a strong high-entropy effect. The SEM and TEM images (Figs. 2f, i and S2) exhibit a uniform cubic shape with an average size of 20 nm. The lattice fringe spacing of 0.288 and 0.292 nm can be assigned to (110) planes of KCoF_3_ (Figs. 2g and S3a) and KCoMnNiMgZnF_3_-HEC (Figs. 2j and S3b), respectively, in good consistent with the XRD results. The inductively coupled plasma-optical emission spectrometer (ICP-OES, Table S4) determines the chemical formula of K_0.95_Co_0.24_Mn_0.20_Ni_0.22_Mg_0.18_Zn_0.16_F_3.05_. The corresponding fast Fourier transform (FFT) patterns (Fig. 2h, k) further confirm the single crystal structures of the products. Energy dispersive X-ray spectroscopy (EDS) mapping images (Fig. 2l, m) present the highly homogeneous elemental distribution, validating the complete formation of single-phase KCoMnNiMgZnF_3_-HEC.

**Fig. 3:**
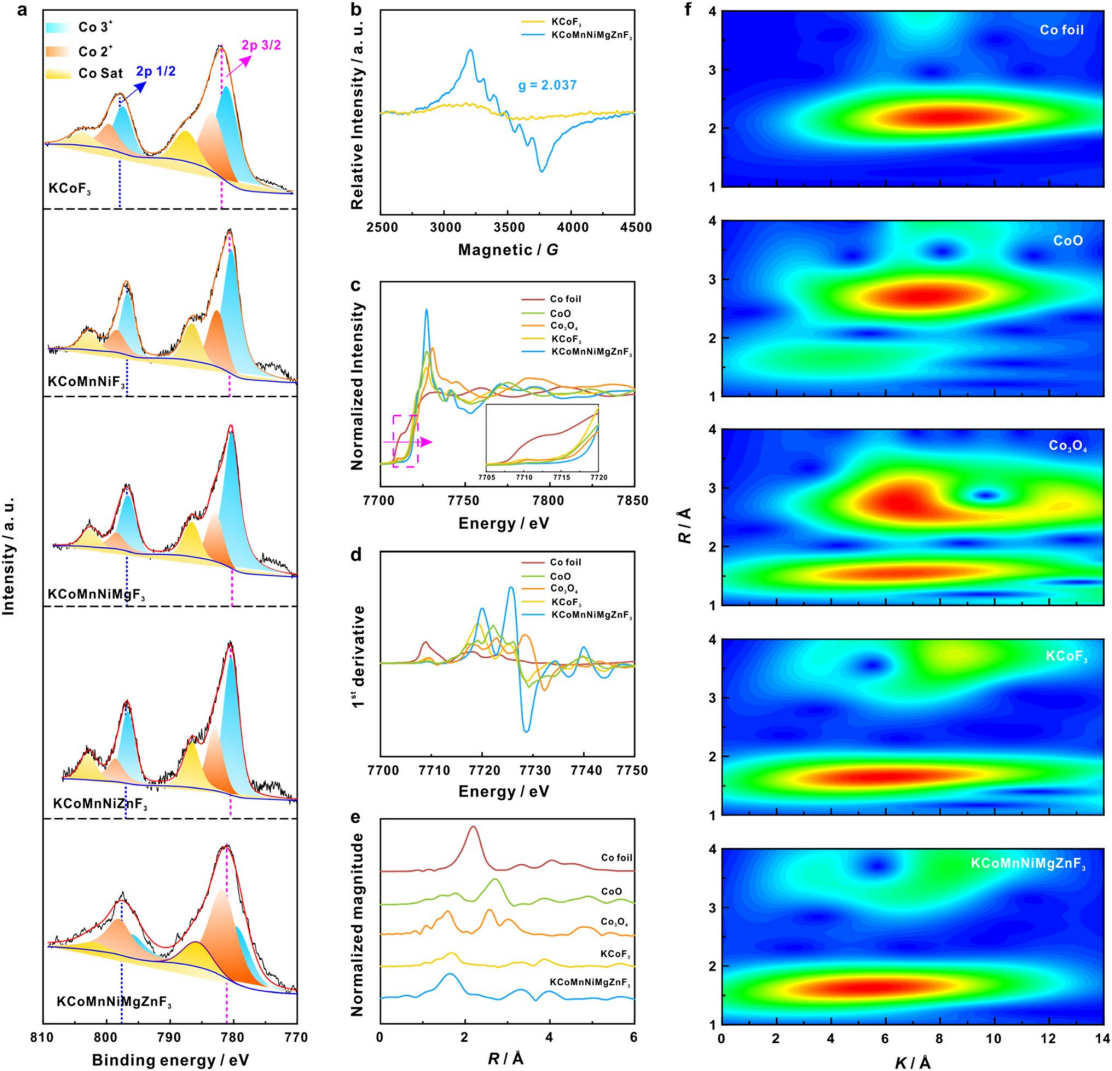
**a** High-resolution Co 2*p* XPS spectra of KCoF_3_, KCoMnNiF_3_, KCoMnNiMgF_3_, KCoMnNiZnF_3_ and KCoMnNiMgZnF_3_-HEC. **b** EPR spectra of KCoF_3_ and KCoMnNiMgZnF_3_-HEC, **c** XANES spectra, **d** first derivatives of XANES and **e** Fourier transformed EXAFS for Co foil, Co_3_O_4_, KCoF_3_, KCoMnNiMgZnF_3_-HEC, and **f** the corresponding WT contour plots

The XPS was carried out to investigate the chemical states and electronic effects of KCoMnNiMgZnF_3_-HEC. Figure S4 demonstrates the presence of these Co, Mn, Ni, Mg, and Zn elements in KCoMnNiMgZnF_3_-HEC. From the Co 2*p* spectrum (Figs. 3a and S5), the main peaks at 780.0, 796.4, 782.2, 798.6, 786.3 and 802.7 eV correspond to Co^3+^ 2*p*_3/2_, Co^3+^ 2*p*_1/2_, Co^2+^ 2*p*_3/2_, Co^2+^ 2*p*_1/2_, Co Sat. 2*p*_3/2_, Co Sat. 2*p*_1/2_, respectively [19]. Compared with KCoF_3_, Co* in KCoMnNiF_3_, KCoMnNiMgF_3_, KCoMnNiZnF_3_, KCoMnNiMgZnF_3_-HEC show negative shifts, indicating the electron donating nature. In comparison, the electron interaction in HEC is most moderate as evidenced by a very minor variation in binding energy. To determine the spin polarization regulation of the as-prepared KCoMnNiMgZnF_3_-HEC, the electron paramagnetic resonance (EPR) spectroscopy was carried out. The major characteristic signals with a *g*-factor of 2.037 (Eq. S1) correspond to the spin Co, Mn, Ni, Mg, Zn species were detected (Figs. 3b and S6), manifesting the presence of unpaired electrons. Meanwhile, the X-ray absorption spectroscopy (XAS) was performed to understand the critical role of high-entropy effect in manipulating the electronic structure of KCoMnNiMgZnF_3_-HEC. X-ray absorption near edge structure (XANES) spectra of Co K-edge (Fig. 3c) reveal a positive oxidation state of Co^2+^ in KCoF_3_ and KCoMnNiMgZnF_3_-HEC. In contrast with KCoF_3_, the peak of derivative XANES of KCoMnNiMgZnF_3_-HEC (Fig. 3d) was shifted to high energy, implying the higher valence state of Co, consistent with the XPS analysis results. In the Fourier-transformed EXAFS spectra (Fig. 3e), KCoMnNiMgZnF_3_-HEC presents only one main peak at ca. 1.65 Å, corresponding to the combined Co-F coordination [20]. This coordination path can be better visualized by the maxima in the wavelet transform (WT) of the EXAFS (Fig. 3f), which is quite different compared to that of Co foil, CoO and Co_3_O_4_. We therefore conclude that the high-entropy effect can modulate the chemical environment and local structure, facilitating the 3*d* electron redistribution and further optimizes *d*-*p* orbital hybridization during electrocatalysis process.

### Evaluation of Electrochemical Performance

**Fig. 4:**
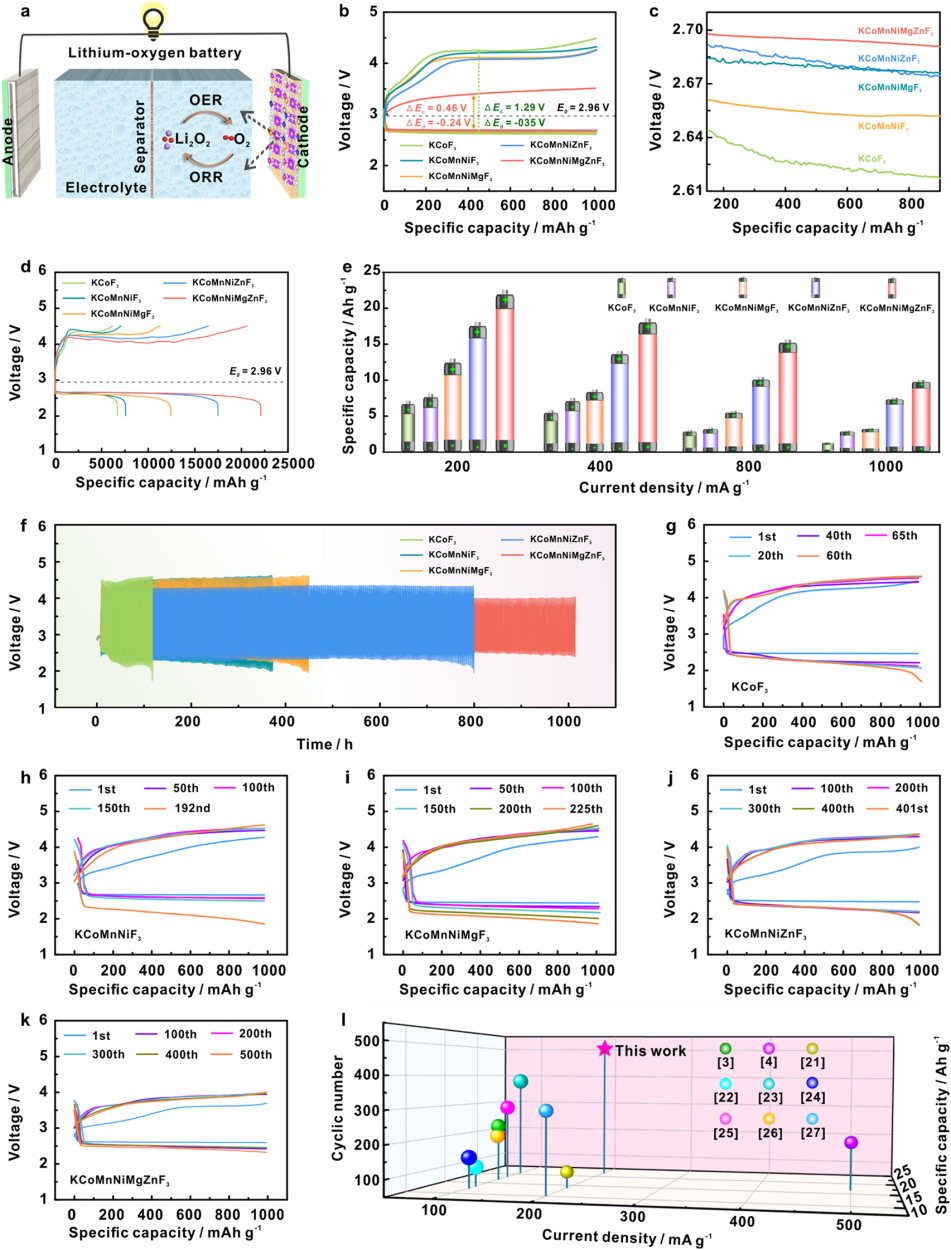
**a** Schematic of a LOB and ORR/OER reaction mechanism. **b** The first discharge–charge voltage curves at a current density of 500 mA g^−1^ under an upper-limit capacity of 1000 mAh g^−1^, and **c** the corresponding details of the selected region in (**b**), **d** the first galvanostatic discharge/charge curves at a current density of 200 mA g^−1^ between 2.0 and 4.5 V, **e** discharge capacities at various current densities, **f** the cycle stability and **g–k** the corresponding galvanostatic discharge/charge curves extracted from whole cycling of the Li–O_2_ cells with KCoF_3_, KCoMnNiF_3_, KCoMnNiMgF_3_, KCoMnNiZnF_3_, and KCoMnNiMgZnF_3_-HEC. **l** Performance comparisons (capacity and cycle stability) of KCoMnNiMgZnF_3_-HEC and other representative published efforts

To make comprehensive analysis of the structure-performance relationship for multi-sites electrocatalyst, the electrocatalytic properties of the as-prepared electrocatalysts were evaluated in a LOB system (Fig. 4a). Figure 4b depicts the first discharge–charge voltage curves at a current density of 500 mA g^−1^ under an upper-limit capacity of 1000 mAh g^−1^. It is evident that the KCoMnNiMgZnF_3_-HEC cathode displays the smallest discharge–charge polarization (0.24/0.46 V), far lower than that of other candidates, illustrating the synergetic action among multiple sites on reaction kinetics of O_2_/Li_2_O_2_ conversion. The corresponding details of ORR process can be visualized in Fig. 4c. In deep discharge–charge process (Fig. 4d), the KCoMnNiMgZnF_3_-HEC cathode delivers an exceptionally high discharge specific capacity of 22,104 mAh g^−1^ at current density of 200 mA g^−1^, much superior to that of KCoF_3_ (6710 mAh g^−1^), KCoMnNiF_3_ (7574 mAh g^−1^), KCoMnNiMgF_3_ (12,423 mAh g^−1^) and KCoMnNiZnF_3_ (17,482 mAh g^−1^). Significantly, the KCoMnNiMgZnF_3_-HEC cathode can maintain its higher capacity retentions and coulombic efficiencies than the other counterparts even at a current density of 400, 800 and 1000 mA g^−1^ (Figs. 4e and S7-S11, Table S5), which corroborates prominent advantage of KCoMnNiMgZnF_3_-HEC in boosting electrochemical performance. Electrochemical impedance spectra (EIS) were performed to survey the reversibility of LOB. As depicted in Fig. S12 (Table S6), the KCoMnNiMgZnF_3_-HEC-based LOB exhibits a low charge transfer resistance (*R*_ct_) at full discharging, indicating a rapid charge transfer process at the three-phase interface of cathode-electrolyte-Li_2_O_2_. Upon recharging, the KCoMnNiMgZnF_3_-HEC-based LOB almost fully recovers to its initial state, which stands in stark contrast to that of KCoF_3_ and highlights its exceptional reversibility.

The cycle life as another critical parameter is measured by long-term discharge/charge test. As revealed in Fig. 4f, the KCoMnNiMgZnF_3_-HEC based LOB achieves a long cycle lifetime of 1000 h (500 cycles) at a high current density of 1000 mA g^−1^ with a limited capacity of 1000 mAh g^−1^, 1.3, 2.2, 2.6, 8.3 times higher than that of KCoF_3_, KCoMnNiF_3_, KCoMnNiMgF_3_, and KCoMnNiZnF_3_, respectively. Figure 4g–k presents the corresponding discharge–charge curves of those candidates during long cycling. It is apparent that the KCoMnNiMgZnF_3_-HEC cathode remains a relatively lower overpotential over long periods, demonstrating extraordinary reversibility. As a result, the notorious parasitic reactions from electrolyte degradation and catalyst poisoning can be effectively suppressed, which contributes to the enhancement of round-trip efficiency and reversibility for LOB. Combining previous theoretical simulations (Fig. 1) and experimental determinations (Fig. 3), the excellent electrochemical performance of KCoMnNiMgZnF_3_-HEC can be attributed to the synergetic action among multiple sites, in which the entropy effect engenders an increased population of unpaired electrons, leading to the electron rearrangement with an elevated spin polarization, dramatically optimizing the *d*-*p* orbital hybridization between cation sites and key intermediate. To our knowledge, those performance parameters of the LOB with KCoMnNiMgZnF_3_-HEC precede majority of traditional catalysts reported in previous studies (Fig. 4l and Table S7) [3, 4, 21–27].

### Evolution of Cathodes Products during Discharge and Charge Process

**Fig. 5:**
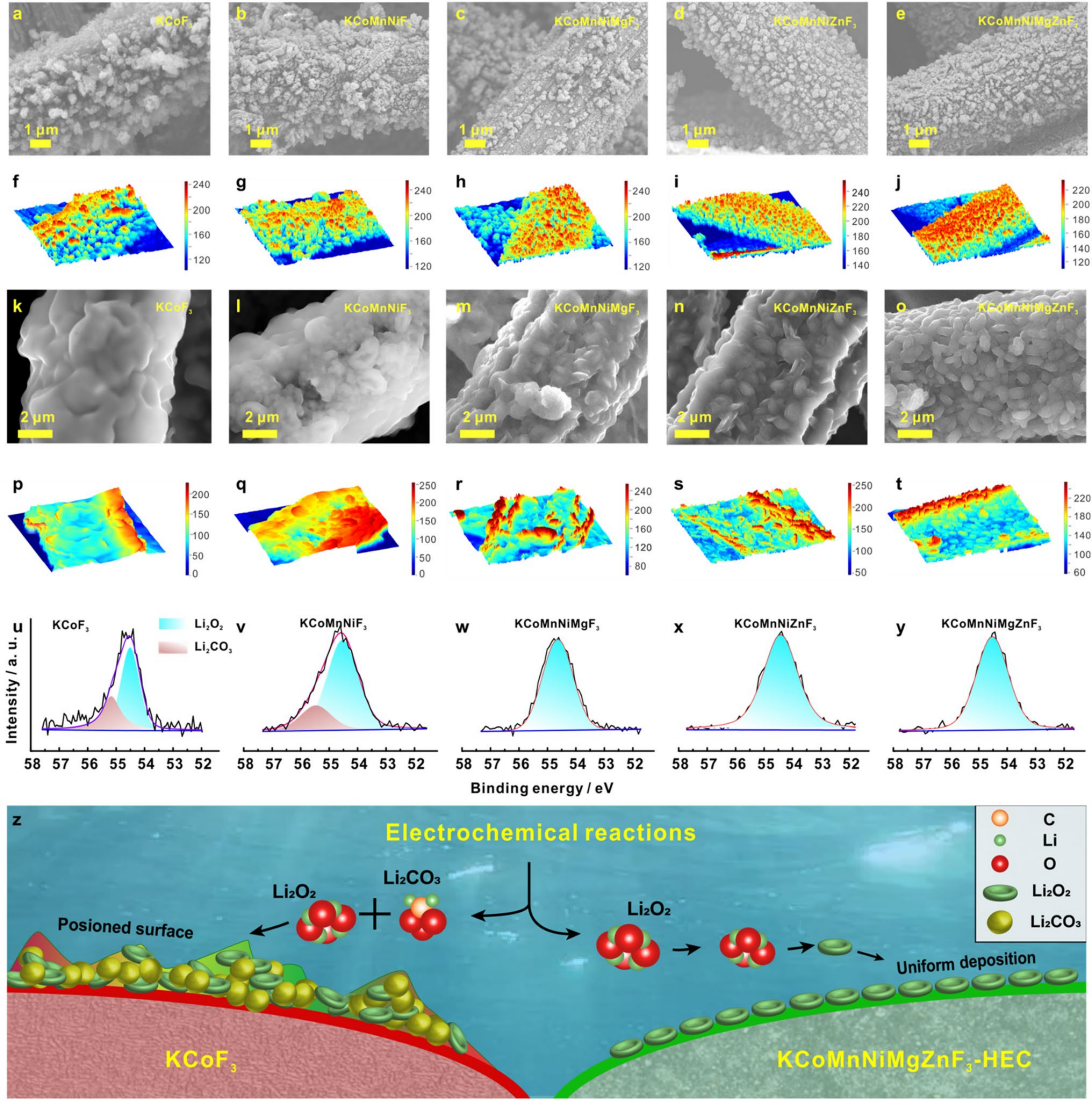
SEM images of cathodes after discharged to 1000 mA h g^−1^ at a current density of 500 mA g.^−1^ with **a** KCoF_3_, **b** KCoMnNiF_3_, **c** KCoMnNiMgF_3_, **d** KCoMnNiZnF_3_, **e** KCoMnNiMgZnF_3_-HEC, and **f–j** The corresponding date visualization. SEM images of cathodes after full discharge with **k** KCoF_3_, **l** KCoMnNiF_3_, **m** KCoMnNiMgF_3_, **n** KCoMnNiZnF_3_, **o** KCoMnNiMgZnF_3_-HEC, and **p–t** The corresponding date visualization. High-resolution Li 1*s* XPS spectra of cathodes after full discharge with **u** KCoF_3_, **v** KCoMnNiF_3_, **w** KCoMnNiMgF_3_, **x** KCoMnNiZnF_3_, **y** KCoMnNiMgZnF_3_-HEC. **z** Schematic illustration of discharge reaction mechanisms on KCoF_3_ (lift) and KCoMnNiMgZnF_3_-HEC (right)

Inspired by the impressive electrochemical performance of KCoMnNiMgZnF_3_-HEC, the morphological evolution and chemical characteristics of cathodes products at various discharge stages was further investigated. Through observation on the SEM images (Fig. 5a–e), it is found that the grain-shaped products appear on those cathodes after discharged to 1000 mAh g^−1^. As revealed in the date visualization (Fig. 5f–j), the distribution of products becomes more and more uniform as the number of metallic elements increment in catalyst, suggesting that the multiple active sites in HEC are capable of promoting the homogeneous nucleation of products. After full discharging, the products on the KCoF_3_ (Fig. 5k, p) and KCoMnNiF_3_ (Fig. 5l, q) cathodes evolve into a closely packed amorphous morphology, which can be identified as Li_2_O_2_ and Li_2_CO_3_ by high-resolution XPS analysis (Fig. 5u, v). In stark contrast, toroidal Li_2_O_2_ accommodations are deposited evenly on the KCoMnNiMgF_3_ (Fig. 5m, r), KCoMnNiZnF_3_ (Fig. 5n, s) and KCoMnNiMgZnF_3_-HEC (Fig. 5o, t) cathode with the absence of obvious signal of Li_2_CO_3_ (Fig. 5w–y). It is worth mentioning that the order degree of cathode products correlated strongly with the number of atomic species in catalyst, verifying the modulating effect of the exposed multiple active sites in HEC on the growth pathway of Li_2_O_2_ (Schematic diagram in Fig. 5z). Essentially speaking, the Li_2_O_2_ morphology and structure are the most important indexes in dominating LOB performance, including overpotential, capacity, cyclability, etc., while the Li_2_O_2_ formation mechanism is influenced by the adsorption behavior of key intermediates, which is closely relevant to the *d*–*p* orbital hybridization between catalyst and LiO_2_. In combination with DFT calculations (Fig. 1i–s), it is reasonable to deduce that mixed elements in KCoMnNiMgZnF_3_-HEC engender significant 3*d* charge redistribution and create multiple active sites with optimized energy barriers for stabilizing LiO_2_ intermediates, which dramatically improves the electrochemical performance of LOB.

**Fig. 6:**
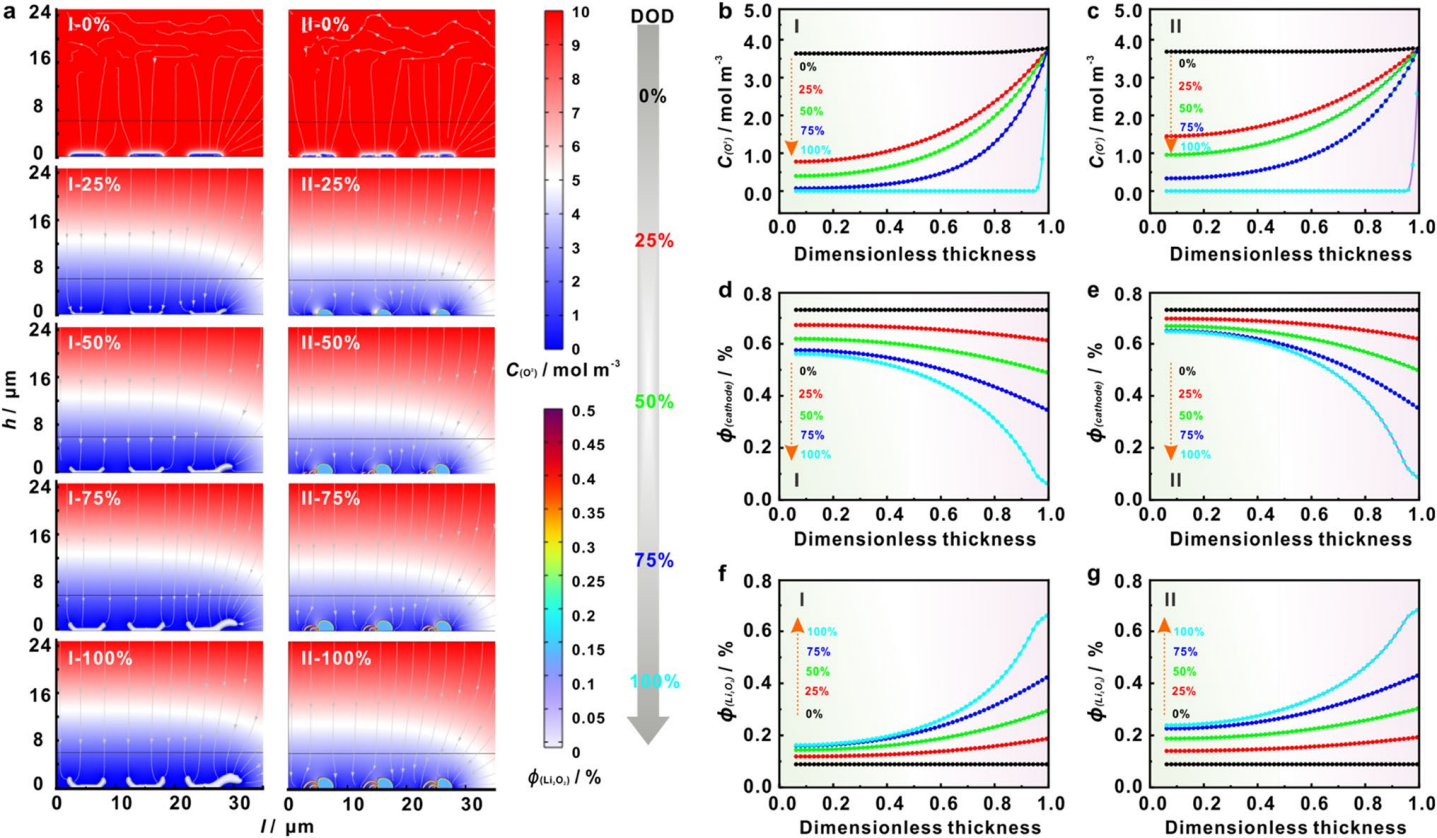
COMSOL simulations of Li_2_O_2_ growth kinetics on cathodes with KCoF_3_ (model I) and KCoMnNiMgZnF_3_-HEC (model II) for **a** O_2_ concentration distributions and Li_2_O_2_ porosity at different discharge stages (0% DOD, 25% DOD, 75% DOD, 100% DOD), **b** and **c** the corresponding curves of O_2_ concentration distributions, **d** and **e** cathode porosity and **f** and **g** Li_2_O_2_ porosity

To thoroughly elucidate the relationship between the Li_2_O_2_ growth model and the electrochemistry toward ORR kinetics, the Li_2_O_2_ deposition behavior on O_2_-electrode was simulated using COMSOL Multiphysics (Fig. S13, Table S8) [28]. Based on Kolmogrov’s phase transformation theory [29] and Sampson and Lynden models [30] combined with experimental results, two 2D transient matrixes were constructed (Figs. S14 and S15). Figure 6a displays the contours of O_2_ concentration distributions and Li_2_O_2_ porosity on O_2_-electrode at different discharge stages, where the heterogeneous nucleation of Li_2_O_2_ on KCoF_3_ cathode donates as model I and homogeneous nucleation of Li_2_O_2_ KCoMnNiMgZnF_3_-HEC cathode donates as model II, taking the previously obtained SEM characterizations (Figs. 5a, k, e, o) as evidence. It is clear that the O_2_ transfer kinetics slows down with the increase of depth of discharge (DOD), which is attributed to the clogging of porous O_2_-electrode due to the accumulation of Li_2_O_2_. We therefore speculate that the formation of O_2_ “dead zone” in cathode was the most common cause of LOB failure. In fact, the accumulation of discharge products on O_2_-electrode cuts off the O_2_-electrode-eletrolyte interaction, which greatly discounts the efficiency of electron and mass transfer, resulting in a constantly-increasing polarization. Videos S1 and S2 exhibited the animate simulations of model I and model II, respectively. From the comparison, the O_2_ diffusion depth, O_2_ concentration (Fig. 6b, c), cathode porosity (Fig. 6d, e) and Li_2_O_2_ porosity (Fig. 6f, g) on model II were significantly higher than that of model I, suggesting that the homogeneous deposition of Li_2_O_2_ triggered by multiple active sites in KCoMnNiMgZnF_3_-HEC can enhance the mass transfer at the three-phase interface among gas-Li_2_O_2_-electrolyte, and thereby alleviating the passivation of O_2_-electrode. Under such circumstance, the LOB enables a fast reaction kinetics, which holds attractive electrochemical performance, well consistent with simulated results (Fig. S16).

### Function of Entropy Effect on Enhanced Cyclic Performance

**Fig. 7:**
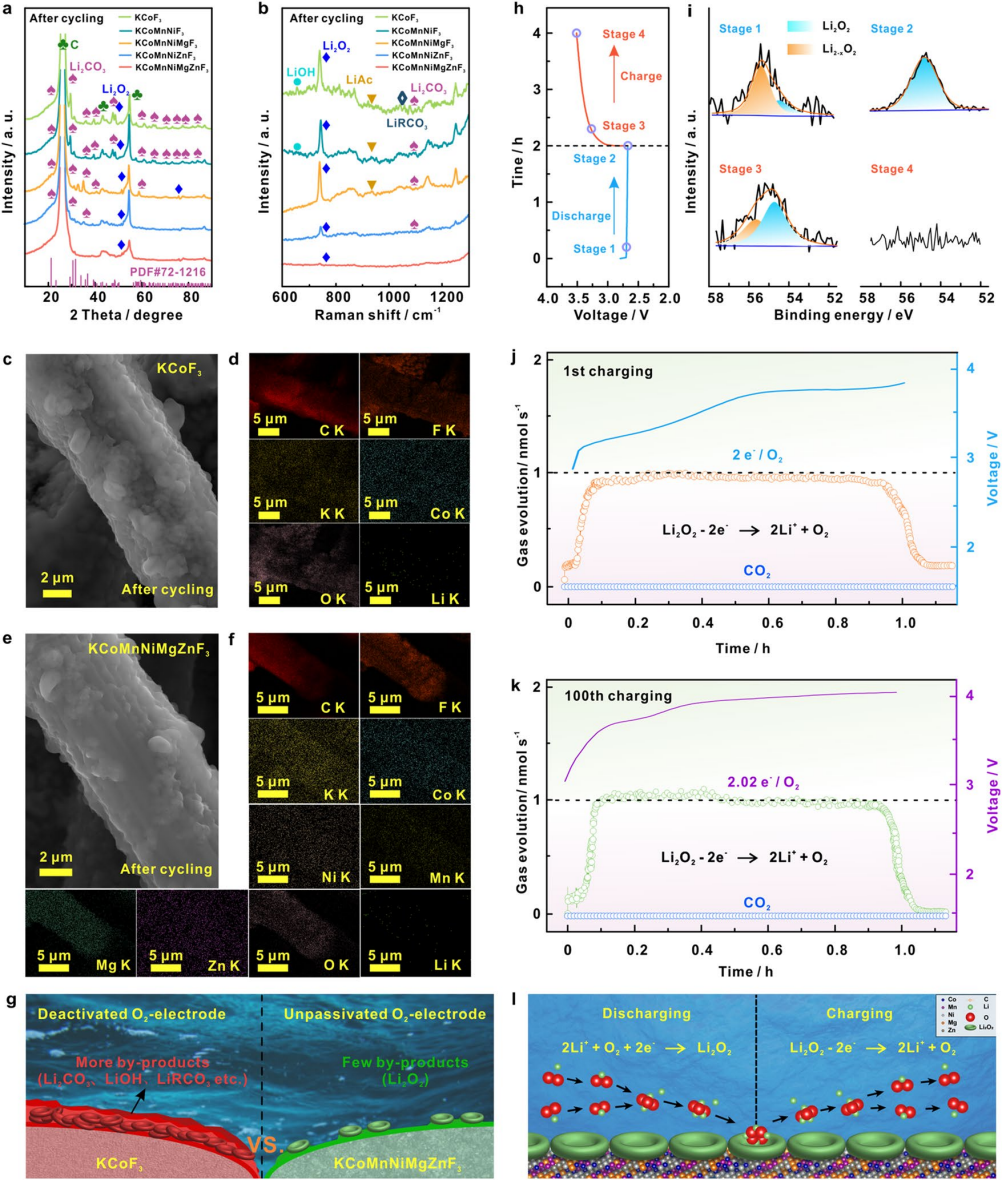
a XRD, and **b** Raman spectra of cathodes with KCoF_3_, KCoMnNiF_3_, KCoMnNiMgF_3_, KCoMnNiZnF_3_, KCoMnNiMgZnF_3_-HEC after cycling at a current density of 1000 mA g^−1^ under an upper-limit capacity of 1000 mAh g^−1^. **c** SEM images and **d** EDS mapping of cathodes after cycling with KCoF_3_. **e** SEM images and **f** EDS mapping of cathodes after cycling with KCoMnNiMgZnF_3_-HEC. **g** Schematic illustration of cycling electrochemistry on KCoF_3_ (lift) and KCoMnNiMgZnF_3_-HEC (right). **h** Galvanostatic discharge–charge curves of the KCoMnNiMgZnF_3_-HEC electrode at a current of 500 mA g^−1^ with an upper-limit capacity of 1000 mAh g^−1^, and **i** high-resolution XPS spectra of Li 1*s* at selected pivotal states as indexed in (**h**). In-situ DEMS curves of LOB with KCoMnNiMgZnF_3_-HEC during charging process for **j** 1st cycling, **k** 100th cycling. **l** Schematic illustration of ORR/OER reaction mechanisms on KCoMnNiMgZnF_3_-HEC cathode

Further investigations were centered on the reversibility of those kinds of cathode. To identify the cathode surface condition, the cycled LOBs were disassembled and analyzed. XRD (Fig. 7a) and Raman patterns (Fig. 7b) reveal the formation of Li_2_CO_3_, LiOH, LiAc, and LiRCO_3_ as main side-products on KCoF_3_, KCoMnNiF_3_, KCoMnNiMgF_3_ and KCoMnNiZnF_3_ cathode [31, 32]. In striking contrast, no signal peak assigned to those byproducts emerges on KCoMnNiMgZnF_3_-HEC cathode, even after 500th cycles, unveiling the suppression effect of multiple active sites in HEC on side reactions. It is clear from Fig. 7c, d that accumulative products completely cover the KCoF_3_ cathode after cycling. Since the attachment of those indissoluble products on cathode surface during cycling, catalytic active centers were gradually blocked, leading to a restricted electrochemistry and aggravated side reactions. In comparison, the KCoMnNiMgZnF_3_-HEC cathode (Fig. 7e, f) basically maintains a fresh interface condition throughout the entire cycles, demonstrating the excellent anti-passivation power. Moreover, the cycled KCoMnNiMgZnF_3_-HEC exhibits a superior preservation of its pristine structure compared to KCoF_3_, as evidenced by Fig. S17, suggesting the excellent stability. Those results provide obvious proof of significant improvement on the long-term durability of LOB with KCoMnNiMgZnF_3_-HEC catalyst. Accordingly, a schematic illustration of the cycling electrochemistry occurring on the KCoF_3_ and KCoMnNiMgZnF_3_-HEC cathode is demonstrated in Fig. 7g.

To determine the reaction pathway and conversion kinetics, high-resolution ex-situ XPS techniques were conducted on the KCoMnNiMgZnF_3_-HEC cathode at selected pivotal states (Fig. 7h). In the Li 1*s* spectra (Fig. 7i), the peaks around 55.9 and 54.8 eV can be assigned to Li_2-x_O_2_ and Li_2_O_2_, respectively. At the beginning of discharge (stage I), the signals of Li_2-x_O_2_ and Li_2_O_2_ appeared in the Li 1*s* spectrum, certifying a multi-step process of ORR. As the discharge proceeded, the ratio of Li_2-x_O_2_ / Li_2_O_2_ decreased significantly (stage II), indicating the transformation of Li_2-x_O_2_ intermediate into Li_2_O_2_ product. Therefore, the possible ORR process on KCoMnNiMgZnF_3_-HEC cathode can be described by the following Eqs. (2–4):

2 $${\text{O}}_{{2}} + e^{ - } {\text{ + Li}}^{ + } \to {\text{LiO}}_{{2}}^{*}$$

3 $${\text{2LiO}}_{{2}}^{* } \to {\text{ Li}}_{{2}} {\text{O}}_{{2}} {\text{ + O}}_{{2}}$$

4 $${\text{LiO}}_{{2}} {* + }e^{ - } {\text{ + Li}}^{ + } \to {\text{Li}}_{{2}} {\text{O}}_{{2}}^{*}$$

When recharged to state III, the intermediate Li_2−*x*_O_2_ reappears on KCoMnNiMgZnF_3_-HEC cathode, implying the de-lithium process of Li_2_O_2_. At the final stage of recharging, the absence of Li 1*s* peak presents the complete decomposition of Li_2_O_2_. The results verify the ability to stabilize key intermediates on multiple active sites in KCoMnNiMgZnF_3_-HEC cathode.

Moreover, the underlying reaction kinetics was analyzed with the assistance of in-situ differential electrochemical mass spectrometry (DEMS). The ratio of 2e^−^/O_2_ on KCoMnNiMgZnF_3_-HEC cathode was displayed in Fig. S18 (Eq. S2), supporting the discharging mechanism involving the reduction of O_2_ along the two-electron pathway (O_2_ + 2Li^+^  + 2e^−^ → Li_2_O_2_) [33]. Notably, during charging process, the oxidation processes were predominantly governed by oxygen releasement, as evidenced by the ratio of 2e^−^/O_2_ in Fig. 7j, suggesting the electrochemical decomposition of Li_2_O_2_ (${\text{Li}}_{{2}} {\text{O}}_{{2 }} {*} - {2 }e^{ - } \to {\text{2 Li}}^{ + } {\text{ + O}}_{{2}}$), without involvement of other side reactions, aligning well with the XRD (Fig. 7a) and Raman (Fig. 7b) results. Due to the stabilization of intermediates on KCoMnNiMgZnF_3_-HEC cathode, two-electron transfer can be achieved most effectively during ORR/OER process, which is of vital importance in boosting reaction kinetics. Encouragingly, the gas analysis plots of O_2_ evolution maintained well in the 100th cycles (Fig. 7k), where the ratio of *v*(e^−^): *v*(O_2_) is determined as 2.02:1, manifesting considerable reversibility of Li_2_O_2_ formation/decomposition on KCoMnNiMgZnF_3_-HEC cathode during long-term cycling. We speculate that the improvement of the durability of LOB is closely correlated to the modulation effect of multiple active sites on the reaction pathway, as illustrated in Fig. 7l.

### Modulation Mechanism of Entropy Effect on Catalytic Kinetics

**Fig. 8:**
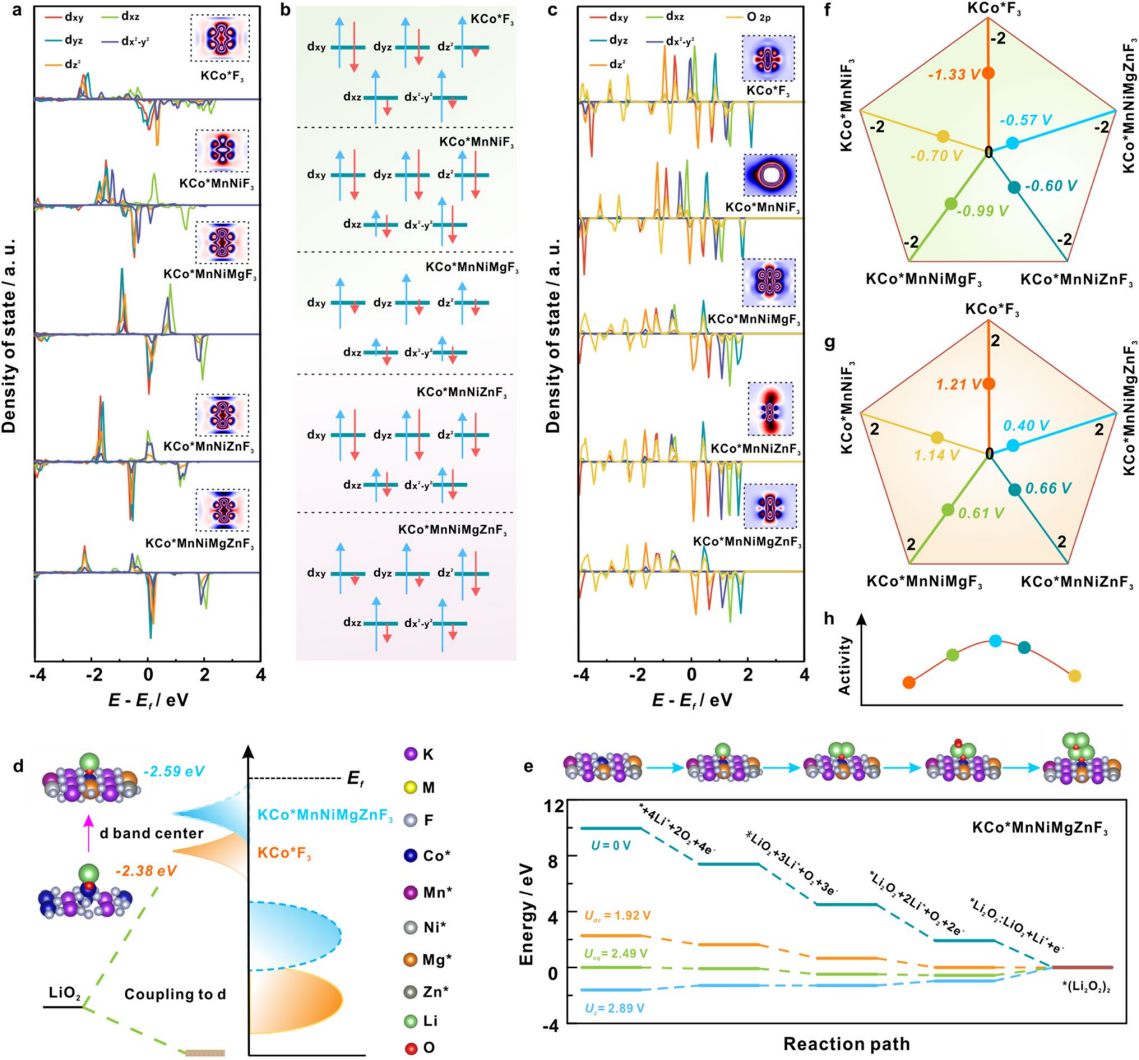
**a** PDOS of Co* 3*d* orbitals, and **b** Schematic diagrams of possible electron arrangements on 3*d* orbital, and **c** PDOS of O 2*p* orbitals for adsorbed LiO_2_ and 3*d* orbitals of Co* centers in KCoF_3_, KCoMnNiF_3_, KCoMnNiMgF_3_, KCoMnNiZnF_3_, KCoMnNiMgZnF_3_-HEC. **d** Schematic illustration of *d*–*p* orbital hybridization during ORR process on Co* site in KCoF_3_ and KCoMnNiMgZnF_3_-HEC. **e** Gibbs free energy diagram of ORR process on Co* site in KCoMnNiMgZnF_3_-HEC. Comparison of **f** ORR overpotentials, **g** OER overpotentials, and **h** catalytic activity among KCoF_3_, KCoMnNiF_3_, KCoMnNiMgF_3_, KCoMnNiZnF_3_, KCoMnNiMgZnF_3_-HEC

To in-depth shed light on the modulation mechanism of entropy effect on catalytic kinetics, we further carried out the DFT simulation and analysis. The local electron configurations of Co* site in KCoF_3_, KCoMnNiF_3_, KCoMnNiMgF_3_ KCoMnNiZnF_3_ and KCoMnNiMgZnF_3_-HEC were first examined through the PDOS calculations. The Co* 3*d* PDOS (Fig. 8a) reveals the rearrangement of 3*d* orbital (*d*_*xz*/*xy*/*yz*_, *d*_*x*_^2^_−*y*_^2^, *d*_*z*_^2^) electron in both spin channels after introducing more metallic elements, signifying the strong interactions among elements. Especially, the *d*_*x*_^2^_−*y*_^2^ and *d*_*z*_^2^ in KCoMnNiMgZnF_3_-HEC leap into Fermi energy level compared to that of KCoF_3_, demonstrating the electron transition from low-energy orbitals to high-energy orbitals [34–36]. Combined with the calculation of *g*-factor (Fig. 3b), it can be concluded that the local charge redistribution increases the unpaired electron density in *d*_*xz*/*xy*/*yz*_ orbitals, enabling the alteration in the spin polarization. The difference charge density maps (the insets of Fig. 8a) present more electrons transfer of Co* in KCoMnNiMgZnF_3_ than that of KCoF_3_, which points to a higher positive valence of Co* in HEC, well consistent with XANES results (Fig. 3c, d). Based on the calculated electron spin states (Tables S9–S13), the possible electron arrangements of Co* in all those candidates were identified, as illustrated in Fig. 8b. Considering the moderate spin polarization of active sites in KCoMnNiMgZnF_3_-HEC, it is believed that the adsorption of key intermediates in O_2_/Li_2_O_2_ conversion can be greatly optimized (Figs. 1j, o and S19), thus bringing about an enhanced intrinsic activity. Base on hybrid orbital theory, the orbital interactions between Co* centers and LiO_2_ intermediate were further discussed. As revealed by Fig. 8c, the *d*_*xz*/*xy*/*yz*_ orbitals in KCoMnNiMgZnF_3_-HEC match better with the 2*p* orbital of adsorbed LiO_2_ in contrast with that of other counterparts, unveiling the optimal *d*-*p* orbital hybridization between HEC and intermediate. Due to the 3*d* orbital coupling of Co* in KCoMnNiMgZnF_3_-HEC, the electron delocalization decreased the *d*_*xz*/*xy*/*yz*_ orbitals energy level, which enables easier electron injection from the Co* *d*_*xz*/*xy*/*yz*_ orbitals into O 2*p* orbital of LiO_2_, thereby facilitating the conversion kinetics. On this basis, the *d*–*p* orbital hybridization during ORR process is visualized in Fig. 8d. In detail, the antibonding orbitals induced by the coupling between O 2*p* and Co* 3*d* located below the Fermi level. Owing to the entropy effect in KCoMnNiMgZnF_3_-HEC, the Co* site denotes a higher *d*-band center and the electron donating nature, which engenders a favorable binding interaction between key intermediate LiO_2_ and Co* site, reinforcing the intrinsic reaction activity.

Figures S19a–d and 8e picturizes the reaction pathways toward the formation of (Li_2_O_2_)_2_ clusters at different overpotentials for the LOBs on Co* site in those various catalysts built on the calculated free energy (Table S14). The discharge and charge overpotentials were referred to evaluate the catalytic activity, defined as ∆*U*_dc_ = *U*_dc_* − U*_eq_, ∆*U*_c_ = *U*_c_* − U*_eq_, where *U*_dc_, *U*_eq_ and *U*_c_ represents the highest discharge potential, equilibrium potential and the lowest charge potential, respectively. It is evident from Fig. 8f–g that the overpotential of KCoMnNiMgZnF_3_-HEC (∆*U*_dc_/∆*U*_c_ = − 0.57/0.40 V) is relatively lower than that of KCoF_3_ (∆*U*_dc_/∆*U*_c_ = − 1.33/1.21 V), KCoMnNiF_3_ (∆*U*_dc_/∆*U*_c_ = − 0.70/1.14 V), KCoMnNiMgF_3_ (∆*U*_dc_/∆*U*_c_ = − 0.99/0.61 V) and KCoMnNiZnF_3_ (∆*U*_dc_/∆*U*_c_ = − 0.60/0.66 V), marking the highest catalytic activity (Fig. 8h). Those DFT results elucidate the modulating effect of the exposed multiple active sites in KCoMnNiMgZnF_3_-HEC on enhancing ORR/OER kinetics, which strongly corroborated the obtained experimental determinations.

## Conclusions

In summary, guided by the DFT screening, we tailored an ideal KCoMnNiMgZnF_3_-HEC catalyst to support the ORR and OER reactions for the development of high-performance LOBs. It is revealed that the entropy effect of multiple sites in KCoMnNiMgZnF_3_-HEC triggers the appropriate regulation of 3*d* orbital structure, leading to a moderate hybridization with the *p* orbital of key intermediates. As a result, KCoMnNiMgZnF_3_-HEC-based LOB exerts an ultrahigh discharge capacity and outstanding long-term cyclability with a lowered overpotentials. Accordingly, we may draw a logical conclusion that, the KCoMnNiMgZnF_3_-HEC catalyst is, momentous to positively manipulate discharge/charge behavior of LOBs. This study demonstrates that the *d* orbital occupancy unidirectionally affects *d*–*p* orbital hybridization between intermediates and catalysts and determines the catalytic kinetics. Tuning the active centers with desirable electron distribution is able to improve the electrochemical performance of LOBs. These findings provide an in-depth understanding of the correlation between multiple active sites in HEC and reaction intermediates, bringing new horizon to the electron modulation and *d*-band center optimization for electrocatalyst.

Although our proposed high-entropy perovskite fluoride catalyst has shown promising results in LOB catalysis, further research is required to fully comprehend the relationship between structural engineering and the optimization of catalytic behaviors, specifically in identifying site function. Furthermore, there is a need to bridge the gap between experimental design and theoretical prediction to achieve seamless connections in the catalytic applications of HEC catalysts. From the long-term point of view, advanced synthesis strategy, high-throughput screening technology and nondestructive characterization are essential for the functionalization of HEC catalysts, both for catalytic purposes and beyond. Through these collaborative efforts, we believe that the materials science and catalysis communities can come together to explore new frontiers in the discovery and design of high-efficient HEC catalysts.

## Supplementary Information

Below is the link to the electronic supplementary material.

Supplementary file1 (PDF 1746 KB)

Supplementary file2 (AVI 907 KB)

Supplementary file3 (AVI 942 KB)
