# Supplementary material for: Enhanced Redox Electrocatalysis in High-Entropy Perovskite Fluorides by Tailoring d–p Hybridization
Source: Nanomicro Lett. 2023 Dec 18;16:55. doi: 10.1007/s40820-023-01275-3 (PMC10728038; doi:10.1007/s40820-023-01275-3)
Supplement: Supplementary file 1 — Supplementary file1 (PDF 1746 KB) [file 40820_2023_1275_MOESM1_ESM.pdf]

Supporting Information for

Enhance Redox Electrocatalysis in High-Entropy Perovskite Fluorides by Tailoring d-p Hybridization

Xudong Li[1], Zhuomin Qiang[2,]*, Guokang Han[2,]*, Shuyun Guan[1], Yang Zhao[1], Shuaifeng Lou[2], and Yongming Zhu[1,]*

[1] Department of Applied Chemistry, Harbin Institute of Technology at Weihai, Weihai 264209, P. R. China

[2] MIIT Key Laboratory of Critical Materials Technology for New Energy Conversion and Storage, School of Chemistry and Chemical Engineering, Harbin Institute of Technology, Harbin 150001, P. R. China

*Corresponding authors. E-mail: 21b925049@stu.hit.edu.cn (Zhuomin Qiang); gkhan@hit.edu.cn (Guokang Han); hitonline@163.com (Yongming Zhu)

Supplementary Tables and Figures

Table S1 Bader charge of Co in $KCoF_3$, Mn in $KMnF_3$, Ni in $KNiF_3$, Mg in $KMgF_3$, Zn in $KZnF_3$, and Co* Mn*, Ni*, Mg*, Zn* in $KCoMnNiMgZnF_3$-HEC based on DFT

	**Bader charge**				
**Candidates**	Co_{133}	Mn_{145}	Ni_{139}	Mg_{156}	Zn_{157}
**Unitary**	7.488962	5.414019	8.490122	8.256795	10.437208
**Quinary**	7.412626	5.235876	8.635671	8.223541	10.587469

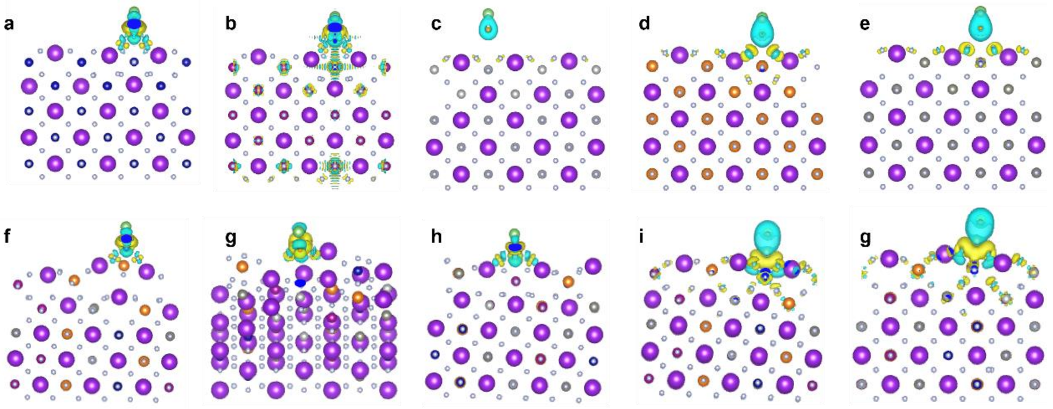

Fig. S1 Optimized structures of intermediate LiO_2 on the sites of **a**) Co in $KCoF_3$, **b**) Mn in $KMnF_3$, **c**) Ni in $KNiF_3$, **d**) Mg in $KMgF_3$, **e**) Zn in $KZnF_3$, and **f**) Co*, **g**) Mn*, **h**) Ni*, **i**) Mg* and **g**) Zn* in $KCoMnNiMgZnF_3$-HEC

Table S2 The Gibbs free energy of each endothermic step through different reaction pathways on site of Co in $KCoF_3$, Mn in $KMnF_3$, Ni in $KNiF_3$, Mg in $KMgF_3$, Zn in $KZnF_3$

Reaction path	**ΔG / eV**				
	**Co**	**Mn**	**Ni**	**Mg**	**Zn**
$* + 4Li^+ + 2O_2 + 4e^-$	8.9247	10.3754	8.1911	16.4693	15.1279
$*LiO_2 + 3Li^+ + O_2 + 3e^-$	6.6004	5.1873	7.4795	14.8067	13.7386
$*Li_2O_2 + 2Li^+ + O_2 + 2e^-$	4.5899	4.0612	3.6997	7.0858	6.6778
$*Li_2O_2{:}LiO_2 + Li^+ + e^-$	1.0253	2.2096	2.2454	4.7208	4.1941
$*(Li_2O_2)_2$	0	0	0	0	0

Table S3 Gibbs free energy of each endothermic step through different reaction pathways on site of Co*, Ni*, Mn*, Mg*, Zn* in $KCoMnNiMgZnF_3$-HEC

Reaction path	**ΔG / eV**				
	**Co***	**Mn***	**Ni***	**Mg***	**Zn***
$* + 4Li^+ + 2O_2 + 4e^-$	9.9505	9.8734	8.876	9.2531	7.1984
$*LiO_2 + 3Li^+ + O_2 + 3e^-$	7.3868	6.7841	8.0505	6.8259	5.9158
$*Li_2O_2 + 2Li^+ + O_2 + 2e^-$	4.4967	4.8806	4.3666	3.8991	3.5352
$*Li_2O_2{:}LiO_2 + Li^+ + e^-$	1.9197	3.0796	1.9669	1.3258	1.1396
$*(Li_2O_2)_2$	0	0	0	0	0

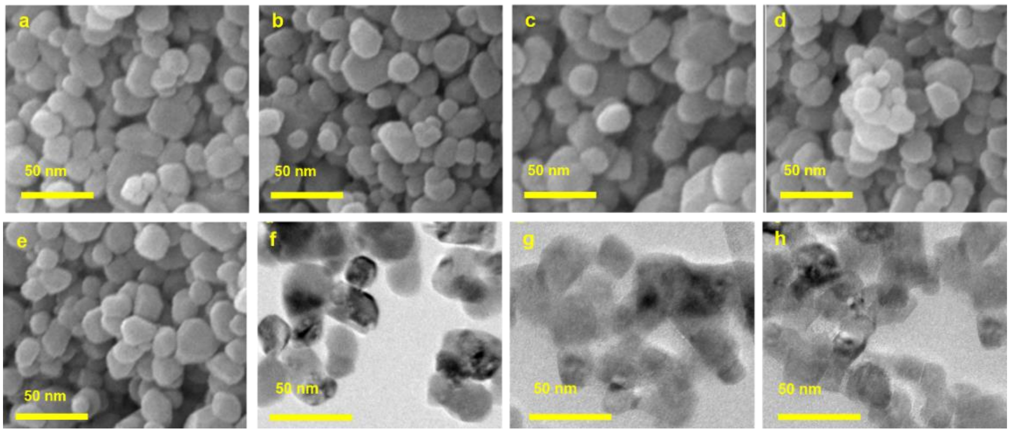

Fig. S2 SEM images of **a**) $KCoF_3$, **b**) $KCoMnNiF_3$, **c**) $KCoMnNiMgF_3$, **d**) $KCoMnNiZnF_3$, **e**) $KCoMnNiMgZnF_3$, and TEM images of **f**) $KCoMnNiF_3$, **g**) $KCoMnNiMgF_3$, **h**) $KCoMnNiZnF_3$

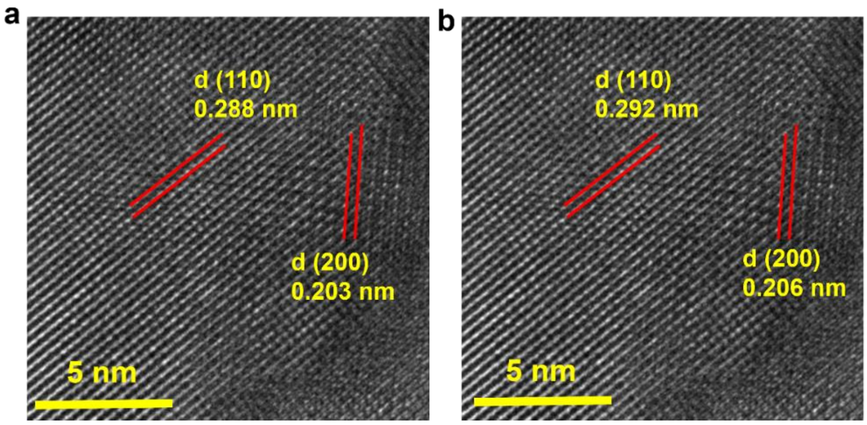

Fig. S3 HRTEM image of a) $KCoF_3$, b) $KCoMnNiZnF_3$

Table S4 Elemental compositions of $K_{0.95}Co_{0.24}Mn_{0.20}Ni_{0.22}Mg_{0.18}Zn_{0.16}F_{3.05}$ determined by ICP-OES analysis.

Mass	K	Co	Mn	Ni	Mg	Zn
Content (mg / L)	37.14	14.10	10.99	12.91	4.19	10.46

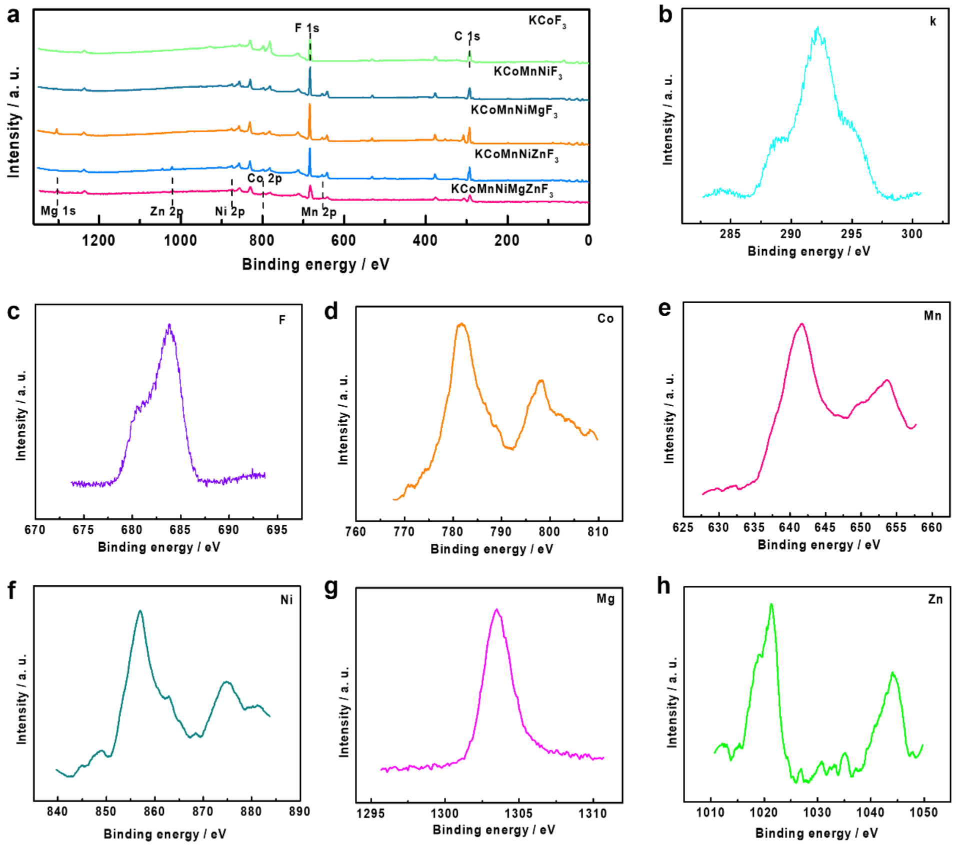

Fig. S4 a) The XPS survey spectra of $KCoF_3$, $KCoMnNiF_3$, $KCoMnNiMgF_3$, $KCoMnNiZnF_3$, and $KCoMnNiMgZnF_3$, and the XPS spectra of K 1*s* **b**), F 2*p* **c**), Co 2*p* **d**), Mn 2*p* **e**), Ni 2*p* **f**), Mg 1*s* **g**), Zn 2*p* **h**) for $KCoMnNiMgZnF_3$

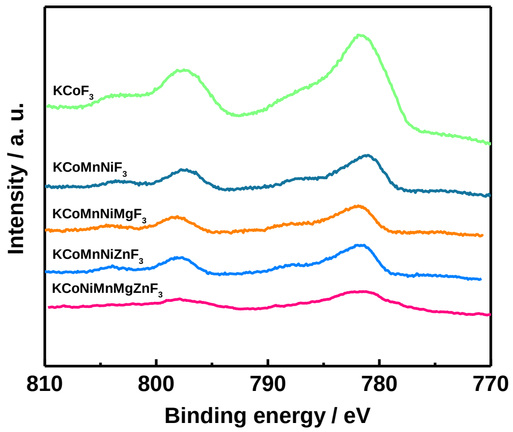

Fig. S5 Co 2*p* XPS spectra of $KCoF_3$, $KCoMnNiF_3$, $KCoMnNiMgF_3$, $KCoMnNiZnF_3$ and KCoMnNiMgZnF

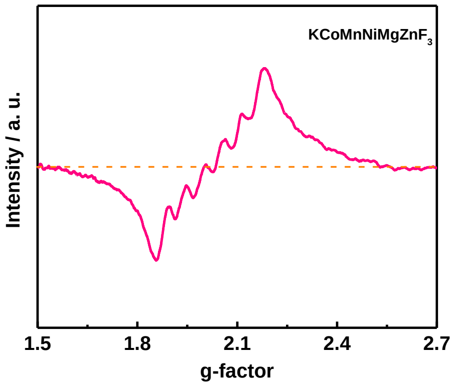

Fig. S6 Calculated g-factor of $KCoMnNiMgZnF_3$ obtained at 25 °C

The g factor was calculated by the following equation:

Equation S1

$g = 71.4477 \times v$ (in GHz) / B (in mT) (S1.1)

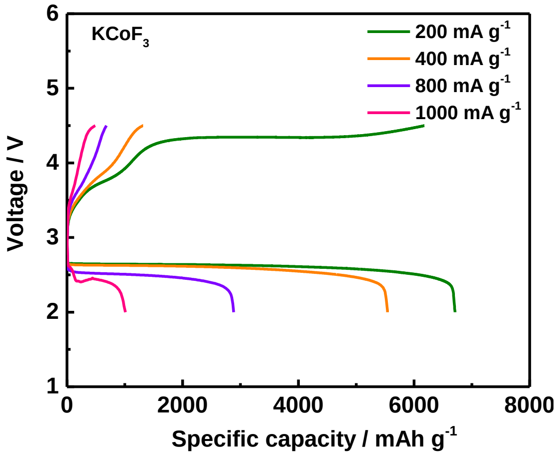

Fig. S7 Galvanostatic discharge/charge curves of LOB with $KCoF_3$ at different current density of 200, 400, 800 and 1000 mA g^{-1} between 2.0 and 4.5 V

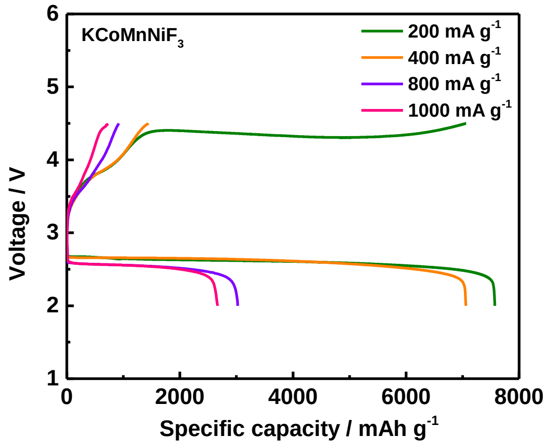

Fig. S8 Galvanostatic discharge/charge curves of LOB with $KCoMnNiF_3$ at different current density of 200, 400, 800 and 1000 mA g^{-1} between 2.0 and 4.5 V

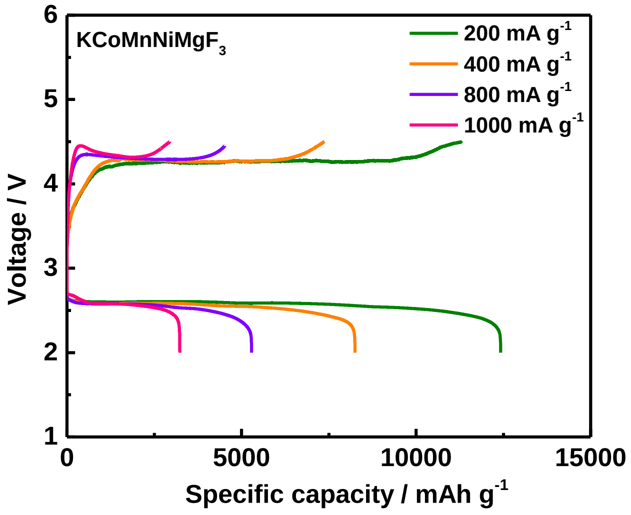

Fig. S9 Galvanostatic discharge/charge curves of LOB with $KCoMnNiMgF_3$ at different current density of 200, 400, 800 and 1000 mA g^{-1} between 2.0 and 4.5 V

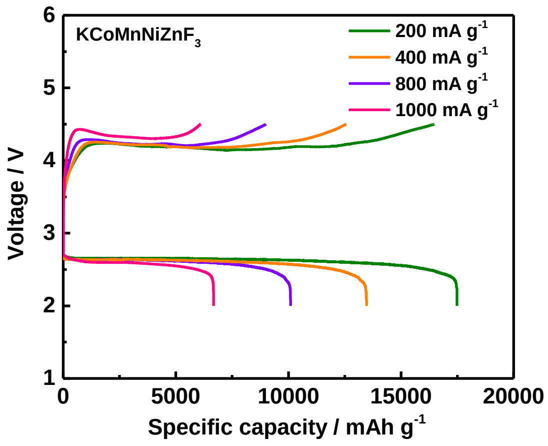

Fig. S10 Galvanostatic discharge/charge curves of LOB with $KCoMnNiZnF_3$ at different current density of 200, 400, 800 and 1000 mA g^{-1} between 2.0 and 4.5 V

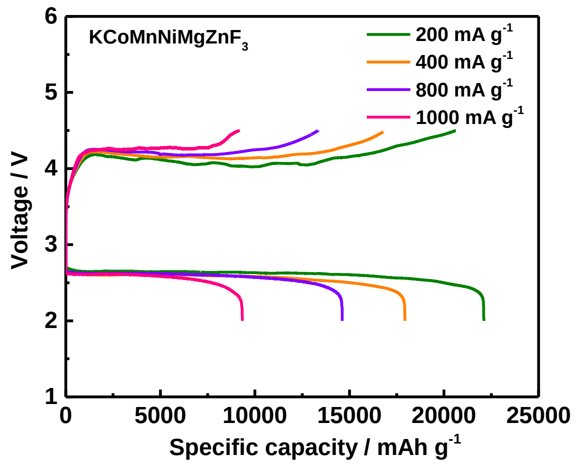

Fig. S11 Galvanostatic discharge/charge curves of LOB with $KCoMnNiMgZnF_3$ at different current density of 200, 400, 800 and 1000 mA g^{-1} between 2.0 and 4.5 V

Table S5 Values of specific capacity at different current density of 200, 400, 800 and 1000 mA g^{-1}

Candidates	**$KCoF_3$**	**$KCoMnNiF_3$**	**$KCoMnNiMgF_3$**	**$KCoMnNiZnF_3$**	**$KCoMnNiMgZnF_3$**
**Current**	**Specific capacity / mAh g^{-1}**				
**200 mA g^{-1}**	6710	7574	12423	17482	22104
**400 mA g^{-1}**	5542	7059	8243	13426	17935
**800 mA g^{-1}**	2880	3026	5284	10075	14617
**1000 mA g^{-1}**	1010	2667	3229	6685	9333

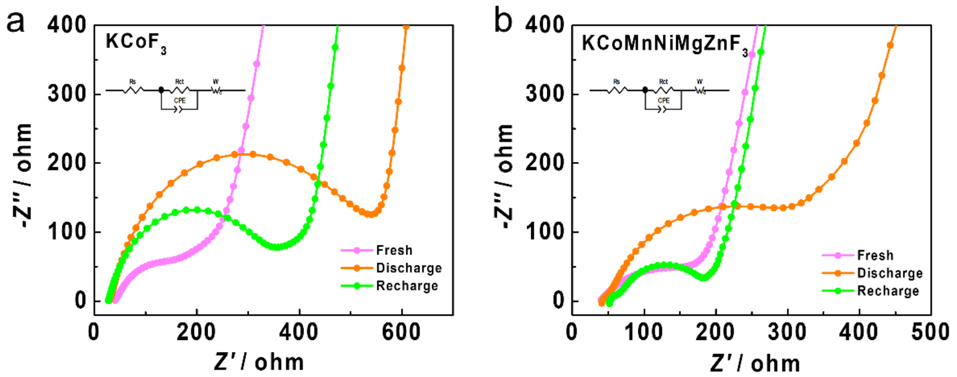

Fig. S12 EIS spectra of LOB with $KCoF_3$ (**a**) and $KCoMnNiMgZnF_3$-HEC (**b**) at different discharge/charge stages

Table S6 The corresponding fitted resistance value at different discharge/charge stages

State	***Fresh***	***Discharge***	***Recharge***	***Δ(Recharge-fresh)***
Candidates	Resistance value / ohm			
**$KCoF_3$**	83.4	427.2	277.8	194.4
**$KCoMnNiMgZnF_3$**	85.8	247.6	116.2	30.4

Table S7 Performance comparison for other representative published efforts

**Materials**	**Current Density ($mA\ g^{-1}$)**	**Specific Capacity ($mA\ h\ g^{-1}$)**	**Upper-limit Capacity ($mA\ h\ g^{-1}$)**	**Stability (cycles)**	**ReferenceS**
NiCoFeO@NF	100	16727	1000	198 cycles at 500 mAg^{-1}	[3]
NiIII-NCF	500	16800	1000	200 cycles at 500 mAg^{-1}	[4]
Pt-CNHS	200	13843	600	100 cycles at 100 mAg^{-1}	[21]
Co[Co, Fe]O_4/NG	100	13312	1000	110 cycles at 100 mAg^{-1}	[22]
2D SnSe	100	20783	600	380 cycles at 500 mAg^{-1}	[23]
CoFeCe	100	12340	1000	145 cycles at 100 mAg^{-1}	[24]
NiS_2/$NiSe_2$	100	18408	600	290 cycles at 200 mAg^{-1}	[25]
$SnSe_2$	100	16871	600	192 cycles at 100 mAg^{-1}	[26]
$NiCo_2S_4$@NiO	200	10050	1000	300 cycles at 200 mAg^{-1}	[27]
**$KCoMnNiMgZnF_3$-HEC**	**200**	**22104**	1000	500 cycles at 1000 mAg^{-1}	**This work**

Computational simulations: Based on COMSOL Multiphysics 6.0 software, we simulated two types of Li_2O_2 deposition behavior on O_2-electrode. The two-dimensional phase field model of the O_2-electrode microenvironment is constructed by coupling different modules of electrochemistry, dilute material transfer, solid mechanics field, domain ordinary differential and differential algebraic equations. In the whole process, considering the anisotropic surface energy, we introduced a thermodynamic framework through data transmission. The operational details were shown as following.

Model assumptions:

(1) Discharge product in porous O_2-electrode only include Li_2O_2.

(2) Li_2O_2 accumulates on the active surface of the porous electrode.

(3) Anode reaction overpotential is ignored.

(4) Temperature changes during discharge process are not considered.

(5) The electrolyte is a uniform organic solvent mixture.

(6) The mass transfer process only include diffusion and electromigration, and ignore convection effect.

(7) The diffusion of Li^+ follows the concentrated solution electrolyte theory.

(8) O_2 is dissolved in the electrolyte with saturated concentration.

(9) The reactant supply is adequate and stable during discharge process.

Establishment of model:

The establishment of mathematical model for electrode microenvironment is based on local assumption principles.

(a) The discharge process is described by the following equation:

$$2Li^{+} + O_2 + 2e^- \rightarrow Li_2O_2 \qquad E_{eq}=2.96\ V \qquad \text{(S4-1)}$$

(b) The electrochemical reaction rate (R_e) is described by the following equation:

$$R_e = -k_0 \tilde{c}_+^{1-\alpha}\ \alpha_{Li2O2}^{\alpha}\ \{exp[\frac{(1-\alpha)nF\eta}{RT}] - exp[\frac{-\alpha nF\eta}{RT}]]\} \qquad \text{(S4-2)}$$

Where k_0 is the reaction rate constant, α_c is the cathode charge transfer coefficient, α_a is the anode charge transfer coefficient, η is the overpotentials, c_i is the concentration, R is the ideal gas constant, T is the temperature, F is Faraday's constant (F= 96487 C/mol), n is the number of electrons transferred.

(c) The overpotential is described by the following equation:

$$\eta = \Delta\phi - \Delta\phi^{eq} = \frac{\Delta\mu}{nF} = \frac{1}{nF}\sum_i \frac{\partial G}{\partial c_i} \qquad \text{(S4-3)}$$

Where Φ is the reaction potential, Φ_{eq} is the reaction equilibrium potential.

(d) The change of phase field is described by the following equation:

$$\frac{\partial\xi}{\partial t} = -k_0 \tilde{c}_+^{1-\alpha} a_M^{\alpha}\{exp[\frac{(1-\alpha)nF_n}{RT}] - exp[\frac{-\alpha nF_n}{RT}]\} \qquad \text{(S4-4)}$$

Where ξ is the phase field, associated with the dimensionless concentration of Li_2O_2.

(e) The effective diffusion coefficient (D^{eff}) is described by the following equation:

$$D^{eff} = D^e h(\xi) + D^s(1 - h(\xi)) \qquad \text{(S4-5)}$$

Where D^e is the diffusion coefficient of cathode, D^s is the diffusion coefficient of

electrolyte

(f) The change of concentration is described by the following equation:

$$\frac{\partial(\varepsilon_1 c_{Li_2O_2})}{\partial(\mathrm{t})} = \frac{-1}{2F} aJ_{loc} \times (c_{Li_2O_2} \geq c_{max,Li_2O_2}) \qquad \text{(S4-6)}$$

Where $c_{max,\ Li2O2}$ is the saturation concentration of Li_2O_2.

(g) The occupation ratio of porosity for Li_2O_2 is described by the following equation:

$$\varepsilon_{Li_2O_2} = 1 - \varepsilon_l - \varepsilon_{s,0} \qquad \text{(S4-7)}$$

(h) The formation rate of Li_2O_2 is described by the following equation:

$$\frac{\partial(\varepsilon_1 c_i)}{\partial(\mathrm{t})} = -\nabla N_i + r_i \qquad \text{(S4-8)}$$

Where N_i is the molar flux, c_i is the volume concentration, r_i is the formation rate from solid to liquid.

The set of coundary conditions:

The established two-dimensional phase field geometric model mainly included porous electrode surface, interfacial double layer and organic electrolyte, as shown in **Fig. S11**. The detailed parameters were listed as following (**Table S7**).

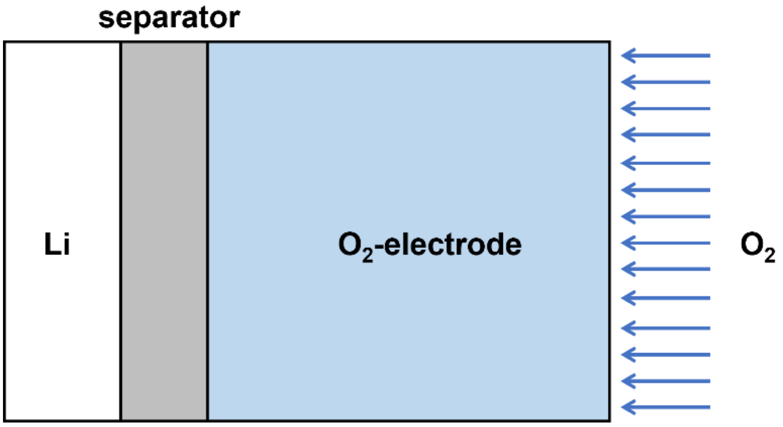

Fig. S13 The established two-dimensional phase field geometric model

Table S8 Parameters in two-dimensional (2D) transient model

Symbol	Value	Parameters
ep	5×10^{-5} m	Thickness of separator
s	7.5×10^{-4} m	Thickness of cathode electrode
K_{pos}	10 S/m	Conductivity of the cathode
ε_0	0.73	Initial porosity of cathode electrode
$\varepsilon_{s,0}$	$1-\varepsilon_0$	Initial active material fraction
epsilonsep	0.87	Porosity of separator
r_{pos0}	25×10^{-9} m	Solid particle radius in cathode

a_{pos0}	$3*\varepsilon_0/r_{pos0}$	Cathode specific surface area
R_{film}	50 $\Omega*m^2$	Electrical resistivity of Li_2O_2
c_{Li0}	1000 mol/m^3	Initial electrolyte salt concentration
Sol_{O2}	0.4	Solubility factor of O_2
c_{O2ext}	9.46 mol/m^3	Reference concentration of O_2
c_{O20}	$Sol_{O2}*c_{O2}ext$	Initial concentration of O_2
$c_{max\ Li2O2}$	0.09 mol/m^3	Li_2O_2 diffusivity in LiTFSI/TEGDME
D_{Li}	2.11×10^{-9} m^2/s	Li^+ diffusion coefficient
D_{O2}	7×10^{-10} m^2/s	diffusion coefficient of O_2
k_{Li}	1.085 S/m	Li^+ conductivity
t^+	0.2594	Li^+ transference number
$d_{lnfdlnc}$	-1.03	$\partial\ lnf / \partial\ lncLi$
ka	1.11×10^{-15} m/s	Reaction rate constant for anode
kc	3.4e-17 $m^7/s/mol^2$	Reaction rate constant for cathode
i_{0refLi}	9.65×10^{-1} A/m^2	Exchange current density for anode
E_{eq}	2.96 V	Equilibrium potential
n	2	Number of transferred electrons
$\rho\ Li_2O_2$	2140 kg/m^3	Mass density of Li_2O_2
$M_{\ Li2O2}$	45.88×10^{-3} kg/mol	Molecular weight of Li_2O_2
T	300 K	Temperature
i_{app}	-0.05*a mA/cm^2	Applied current density
L_σ	1×10^{-6}（m^3/Js）	Interfacial mobility
L_η	0.5（s）	Reaction constant
κ	5×10^{-5}（J/m）	Gradient energy coefficient
γ	0.6（J/m^2）	Interfacial energy
W	1×10^5（J/m^3）	Reaction energy barrier
l	500µm	Length
Δt	0.2（s）	Time interval
D^e	7.5×10^{-13}（m^2/s）	Ionic diffusion coefficient of electrode
D^s	7.5×10^{-10}（m^2/s）	Ionic diffusion coefficient of solution
σ^e	1×10^7（S/m）	Electrode conductivity
σ^S	0.1（S/m）	Solution conductivity

Grid division of two models: In COMSOL Multiphysics, the model grid was set up by controlling the physical field. The model was mesh-divided though a free triangle mesh, in which the mesh precision was ultra-refined, contaning 139028 domain units and 1822 boundary units. The errors at the grid level were controlled within the acceptable range. According to the distribution of catalytic site distributions and morphology of intial Li_2O_2 (**Fig. 5a, e**), we built two types of grid division, as shown in **Fig. S14** (model I) and **Fig. S15** (model II).

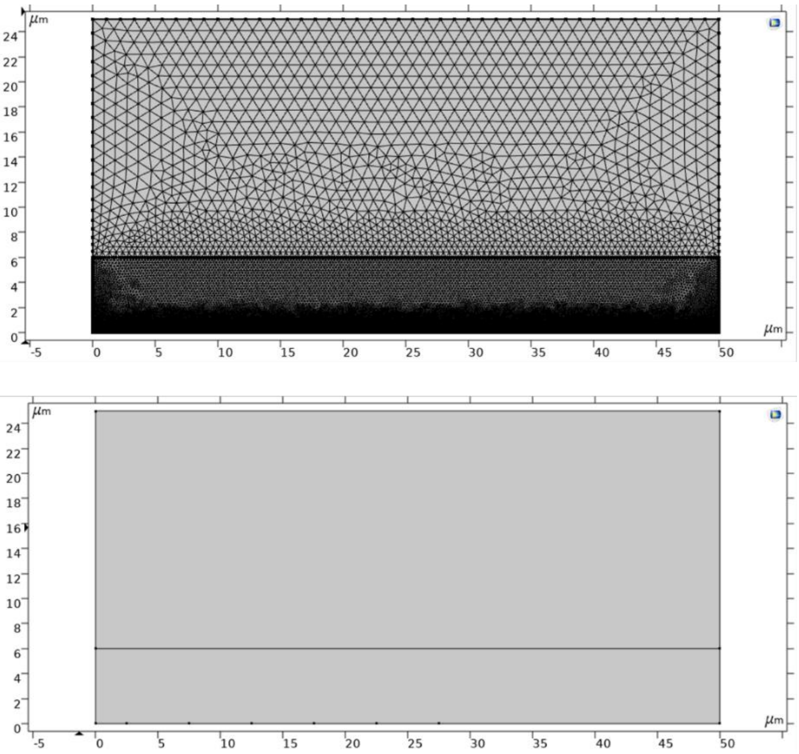

Fig. S14 The grid division structure diagram of model I with $KCoF_3$

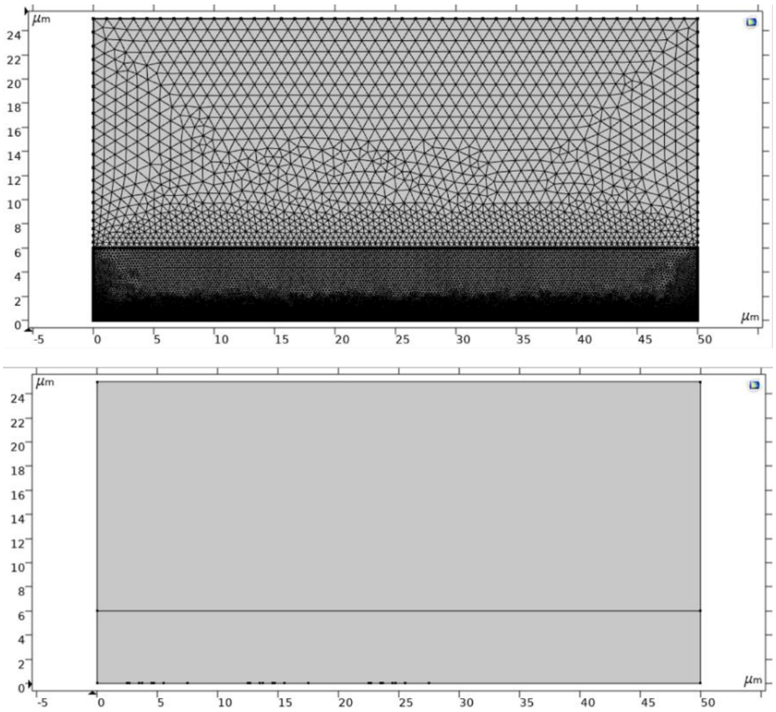

Fig. S15 The grid division structure diagram of model II $KCoNiMnMgZnF_3$-HEC

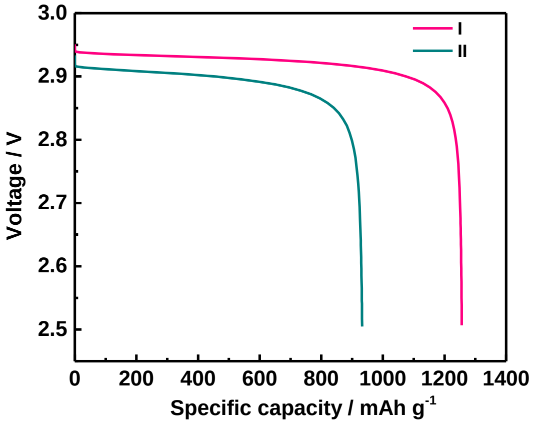

Fig. S16 Simulative discharge performance of LOB with those two models

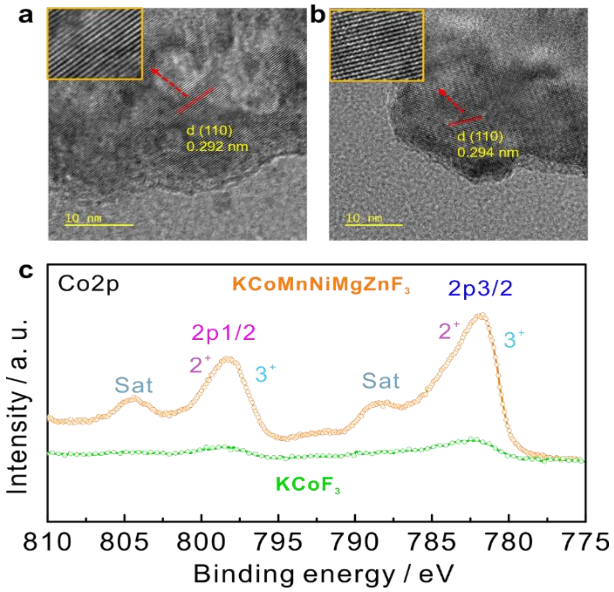

Fig. S17 SEM images of the $KCoF_3$ (a) and HEC (b) after cycling, and the corresponding high-resolution Co 2p XPS spectra (c)

In-situ DEMS measurement: A customized Swagelok-type Li-O_2 cell was used in this study, with two PEEK capillary tubes serving as purge gas inlet and outlet. A specially-designed gas-purging system was utilized to connect the Li-O_2 cell to a commercial magnetic sector mass spectrometer (Qulee, QCS-ULVAC). The flow rate of purge gas was controlled by a digital mass flow meter (Bronkhorst). During the discharging process, a mixture of Ar/O_2 (molar ratio 1/4) with a flux of 5 mL min^{-1} was used as the working gas for the purpose of quantifying O_2 consumption. During the discharging process, high-purity Ar was used as the carrier gas. In either case, Ar was included as an internal trace gas with a known invariable flux to ensure accurate measurement. The DEMS cell assembly resembled that mentioned in the electrochemical measurements part. In terms of the discharge and charge conditions, all cells were discharged/charged at a constant current of 1000 mA g^{-1}, with the capacity being limited to 1000 mAh g^{-1}. These parameters were carefully selected to ensure reliable and consistent performance in the Li-O_2 cells, facilitating accurate characterization of the electrochemical reactions.

The ratio of transferred charge to O_2 production was calculated by the following equation:

Equation S2

$$Q = I \times t \quad \text{(S2.1)}$$

$$v\,(e^-) = Q \times e^{-1} \quad \text{(S2.2)}$$

$$v\,(O_2) = \text{GasEvol.Rate} / 60 \times \text{NA} \quad \text{(S2.3)}$$

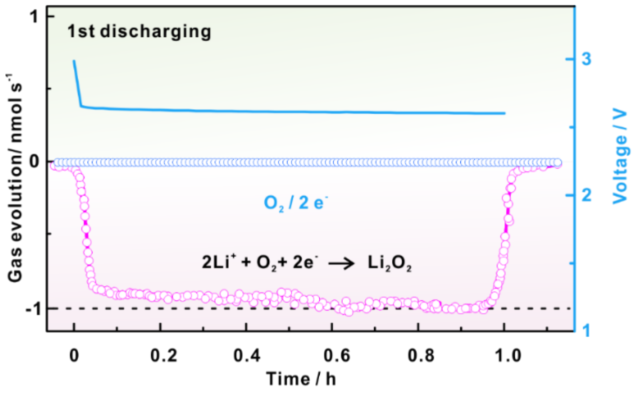

Fig. S18 In-situ DEMS curves of LOB with $KCoMnNiMgZnF_3$-HEC during 1st discharging process

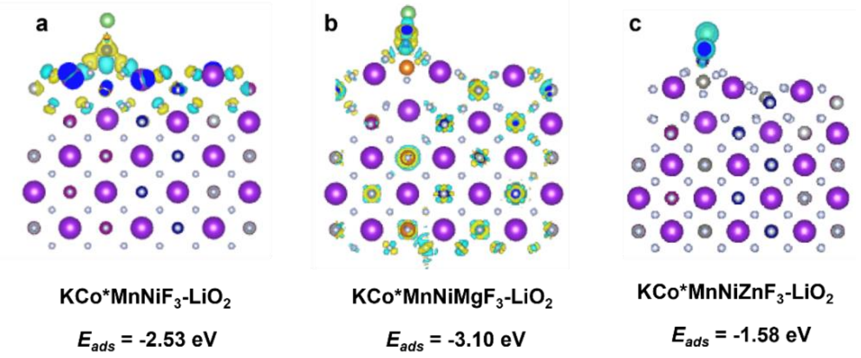

Fig. S19 Optimized structures and the corresponding binding energy of intermediate LiO_2 on the sites of a) Co* in $KCoMnNiF_3$, b) Co* in $KCoMnNiMgF_3$, c) Co* in $KCoMnNiZnF_3$

Table S9 The calculated electron spin states of $KCoF_3$

Electron spin state	**KCo^*F_3**				
	d_{xz}	d_{xy}	d_{yz}	$d_{x^2-y^2}$	d_{z^2}
spin-up	1.04037	0.97233	0.97003	1.00091	0.98341
spin-down	-0.31946	-0.96399	-0.68741	-0.22961	-0.18652

Table S10 The calculated electron spin states of $KCoMnNiF_3$

Electron spin state	**$KCo^{*}NiMnF_3$**				
	d_{xz}	d_{xy}	d_{yz}	$d_{x^2-y^2}$	d_{z^2}
spin-up	0.51891	0.96459	0.96272	0.94011	0.96284
spin-down	-0.43218	-0.9616	-0.95785	-0.71925	-0.91396

Table S11 The calculated electron spin states of $KCoMnNiMgF_3$

Electron spin state	**$KCo^{*}NiMnMgF_3$**				
	d_{xz}	d_{xy}	d_{yz}	$d_{x^2-y^2}$	d_{z^2}
spin-up	0.34831	0.97412	0.97309	0.47502	0.81354
spin-down	-0.3651	-0.2482	-0.30417	-0.34011	-0.32597

Table S12 The calculated electron spin states of $KCoMnNiZnF_3$

Electron spin state	**$KCo^{*}NiMnZnF_3$**				
	d_{xz}	d_{xy}	d_{yz}	$d_{x^2-y^2}$	d_{z^2}
spin-up	0.65667	0.97406	0.97596	0.69187	0.87386
spin-down	-0.47293	-0.97785	-0.97288	-0.58487	-0.8445

Table S13 The calculated electron spin states of $KCoMnNiMgZnF_3$-HEC

Electron spin state	**$KCo^{*}NiMnMgZnF_3$**				
	d_{xz}	d_{xy}	d_{yz}	$d_{x^2-y^2}$	d_{z^2}
spin-up	1.04931	0.97962	0.97806	1.02605	0.99481
spin-down	-0.36372	-0.21378	-0.25812	-0.32003	0.86207

Table S14 The Gibbs free energy of each endothermic step through different reaction pathways on Co sites in $KCoF_3$, $KCoMnNiF_3$, $KCoMnNiMgF_3$, $KCoMnNiZnF_3$, $KCoMnNiMgZnF_3$-HEC

Reaction path	**Δ*G* / eV**				
	**$KCo^{*}F_3$**	**$KCo^{*}NiMnF_3$**	**$KCo^{*}NiMnMgF_3$**	**$KCo^{*}NiMnZnF_3$**	**$KCo^{*}NiMnMgZnF_3$**
$* + 4Li^{+} + 2O_2 + 4e^{-}$	8.9247	8.2651	9.4732	8.6371	9.9505
$*LiO_2 + 3Li^{+} + O_2 + 3e^{-}$	6.6004	5.7599	6.4025	7.0808	7.3868
$*Li_2O_2 + 2Li^{+} + O_2 + 2e^{-}$	4.5899	3.8158	3.3475	3.6802	4.4967
$*Li_2O_2{:}LiO_2 + Li^{+} + e^{-}$	1.0253	2.4536	1.9418	2.2517	1.9197
$*(Li_2O_2)_2$	0	0	0	0	0

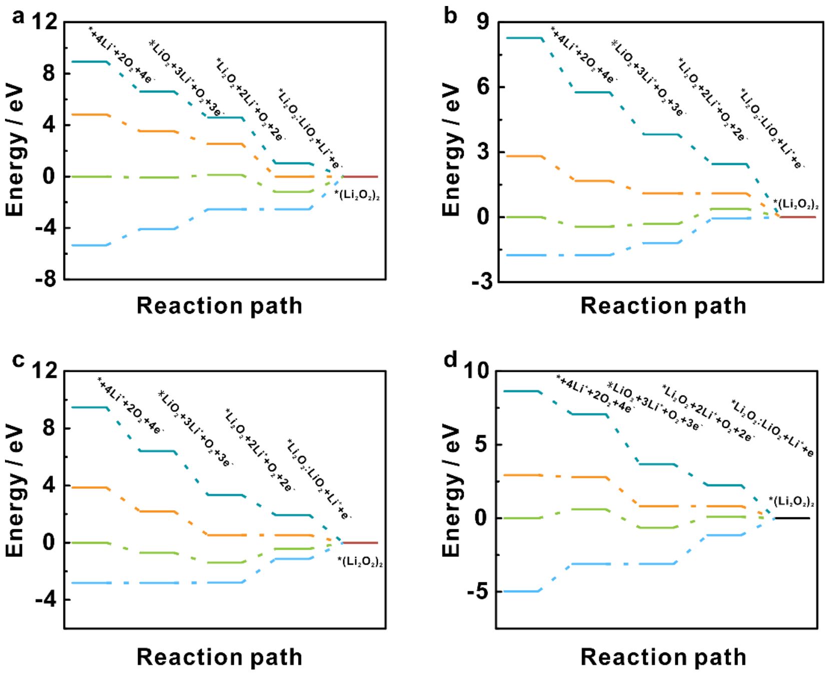

Fig. S20 Gibbs free energy diagram of ORR process on Co* site in **a**) $KCoF_3$, **b**) $KCoMnNiF_3$, **c**) $KCoMnNiMgF_3$, **d**) $KCoMnNiZnF_3$
